# Erbium-substituted LaFeO_3_ perovskite nanoparticles for pseudocapacitive energy storage applications

**DOI:** 10.1039/d6ra04225b

**Published:** 2026-08-05

**Authors:** Tomal Saha†, Ananya Roy, Sadman Yasir, Shahid Bin Anik, Md. Fakhrul Islam, Aninda Nafis Ahmed, Takian Fakhrul

**Affiliations:** a Department of Materials and Metallurgical Engineering, Bangladesh University of Engineering and Technology (BUET) Dhaka 1000 Bangladesh takianf@mme.buet.ac.bd; b Department of Nanomaterials and Ceramic Engineering, Bangladesh University of Engineering and Technology (BUET) Dhaka 1000 Bangladesh; c Pilot Plant and Process Development Centre, Bangladesh Council of Scientific and Industrial Research (BCSIR) Dhaka 1205 Bangladesh a_nafis_ahmed@bcsir.gov.bd

## Abstract

LaFeO_3_ perovskites are promising candidates for supercapacitor electrodes, yet their performance is limited by low ionic conductivity and restricted active surface area. In this work, Er^3+^ was introduced at the A-site of LaFeO_3_ to increase defect concentration and improve ion transport within the perovskite lattice. Nanoparticles with compositions of La_1−*x*_Er_*x*_FeO_3_ (*x* = 0, 0.1, 0.2, and 0.3) were synthesized using a sol–gel auto-combustion method and characterized by XRD, SEM, TEM, and XPS to establish structural refinement, morphological evolution, and defect chemistry. Among all compositions, La_0.8_Er_0.2_FeO_3_ delivered the highest specific capacitance of 288.2 F g^−1^ at 5 mV s^−1^ and 201.4 F g^−1^ at 2 A g^−1^. XPS revealed a significant increase in defect concentration, accompanied by the formation of Fe^2+^*via* electron transfer from O^2−^ ions, consistent with enhanced defect-assisted charge transport. Shortened oxygen diffusion pathways arising from reduced bond lengths further contributed to improved ionic mobility. The Ragone analysis indicated that the Er-doped composition exhibits superior energy and power densities compared to pristine LaFeO_3_. These results establish Er substitution as an effective strategy for defect engineering in LaFeO_3_, enabling improved charge-storage behavior and positioning Er-doped LaFeO_3_ as a strong candidate for next-generation pseudocapacitive energy-storage applications.

## Abstract 2

Er^3+^-induced defect engineering in LaFeO_3_ enhances ion transport, shortens diffusion pathways, and improves pseudocapacitive charge storage, achieving a maximum capacitance of 288.2 F g^−1^ in La_0.8_Er_0.2_FeO_3_.

## Graphical abstract

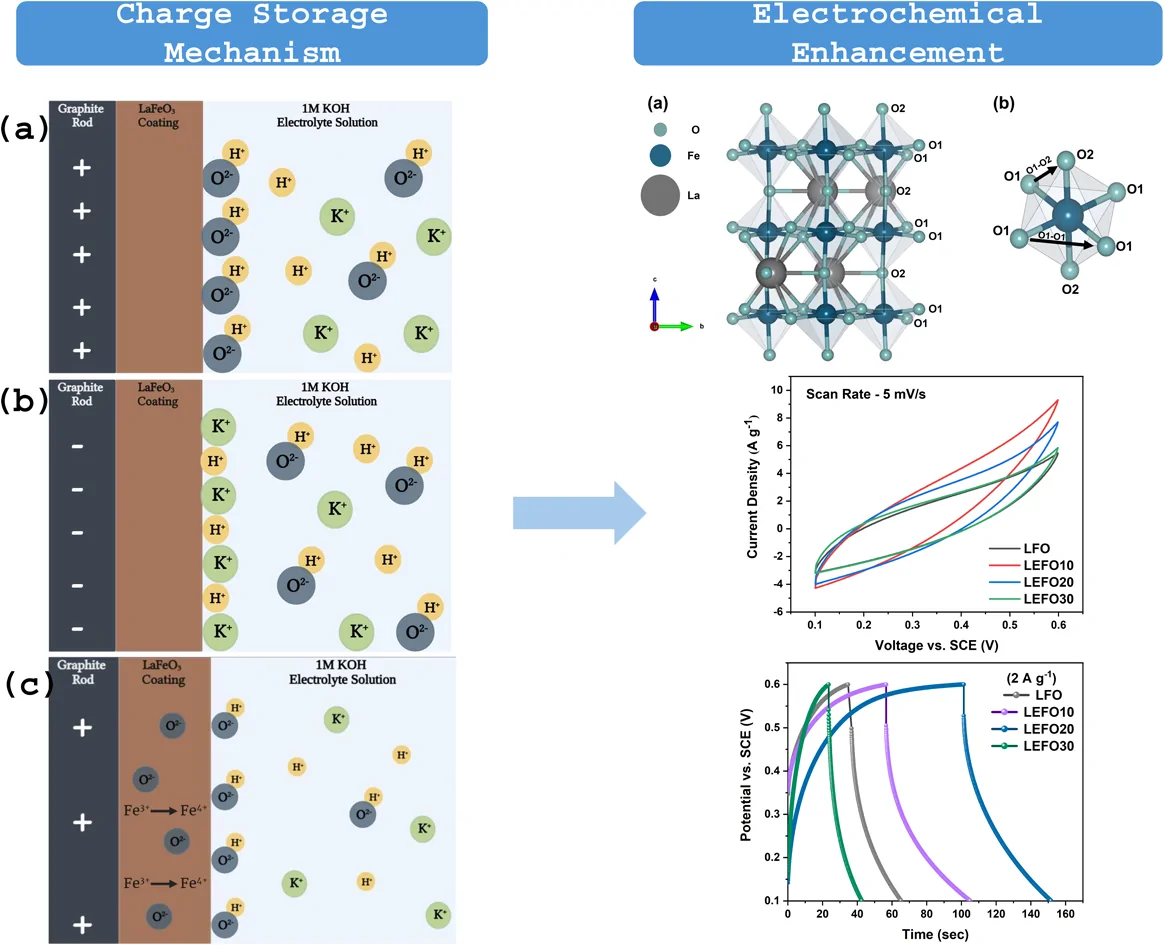

## 1. Introduction

Perovskite oxides, with the general formula ABO_3_, have attracted extensive interest due to their structural versatility, defect tunability, and broad applicability in energy conversion and storage. Their multifunctional behavior enables the design of advanced materials for gas sensing,^1^ catalytic activity,^2,3^ solid oxide fuel cells, and supercapacitors.^4–7^ Among transition metal-based perovskites, LaFeO_3_ has emerged as a compelling system for pseudocapacitive charge storage because of its thermal stability, low cost,^8^ wide operational potential window,^9^ and rich defect chemistry. The inherent presence of oxygen vacancies,^8,10^ multiple oxidation states of Fe,^11^ and favorable surface oxygen exchange kinetics^12^ provide a strong foundation for redox-based energy-storage mechanisms.

Supercapacitors operate through either electrical double-layer capacitance or pseudocapacitance. Pseudocapacitive transition metal oxides, including perovskite ferrites, store charge through faradaic surface reactions^13^ or through bulk intercalation processes.^10^ In perovskite oxides, oxygen vacancies play a critical role in enabling rapid redox activity and facilitating oxide-ion mobility.^14,15^ Increasing the oxygen vacancy concentration,^12,16^ reducing particle size,^17^ and shortening diffusion pathways are therefore effective strategies for improving electrochemical performance.

Although LaFeO_3_ possesses intrinsic redox activity, its practical capacitance is often limited by particle agglomeration, low ionic conductivity, and restricted electrochemical surface area.^18^ To overcome these challenges, compositional modification through A- or B-site substitution has been widely explored. Previous studies have demonstrated that doping with Zn,^19^ Al,^20^ or Sr^21^ can significantly increase the specific capacitance by tuning lattice strain, modifying Fe valence states, and enhancing defect populations. In contrast, investigations on the role of rare-earth substitution at the A-site of LaFeO_3_ for energy storage applications remain very limited. The only closely related report involves co-doped Nd-Mn LaFeO_3_ and its subsequent composite formation with nitrogen-doped graphene oxide,^22^ where the electrochemical behavior is governed by the combined effects of co-doping and heterostructure formation rather than the independent influence of rare-earth substitution. As a result, the intrinsic impact of rare-earth dopants on defect chemistry, oxygen vacancy generation, and pseudocapacitive charge-storage behavior in LaFeO_3_ has not yet been clearly established.

Erbium offers a promising dopant for defect engineering in LaFeO_3_. The smaller ionic radius of Er^3+^ compared with that of La^3+^ introduces local lattice distortion, suppresses grain growth,^17^ and increases the concentration of oxygen vacancies.^1,23^ These structural and chemical modifications are expected to enhance ion diffusion,^14,15^ improve charge-transfer kinetics,^15^ and strengthen pseudocapacitive behavior. Despite these advantages, Er-doped LaFeO_3_ has not yet been systematically evaluated for supercapacitor applications.

In this work, La_1−*x*_Er_*x*_FeO_3_ (*x* = 0, 0.1, 0.2, and 0.3) nanoparticles were synthesized using a sol–gel auto-combustion method to investigate the influence of Er substitution on structure, morphology, defect chemistry, and electrochemical performance. Comprehensive characterization through XRD, Rietveld refinement, SEM, TEM, and XPS was conducted to elucidate dopant-induced transformations. Electrochemical testing, including cyclic voltammetry, galvanostatic charge–discharge, and impedance spectroscopy, was used to establish the relationship between defect evolution and charge-storage behavior.

This study demonstrates that Er substitution is an effective route for tuning defect concentration and improving pseudocapacitive performance in LaFeO_3_. To the best of our knowledge, a systematic investigation of Er substitution in LaFeO_3_ for pseudocapacitive energy storage, with explicit correlation between defect chemistry and electrochemical performance, has not been reported. The findings provide new insight into rare-earth-based defect engineering strategies for advanced perovskite energy-storage materials beyond conventional alkaline-earth or transition-metal doping.

## 2. Experimental

### 2.1 Materials

Erbium nitrate pentahydrate [Er(NO_3_)_3_·5H_2_O > 99.0%], lanthanum nitrate hexahydrate [La(NO_3_)_3_·6H_2_O > 99.0%] and iron nitrate nonahydrate [Fe(NO_3_)_3_·9H_2_O > 98.0%] were used as precursors. Citric acid monohydrate (C_6_H_8_O_7_·H_2_O), ethylene glycol (C_2_H_6_O_2_), and DI (de-ionized) water were employed as chelating agent, stabilizing agent, and solvent, respectively. The chemicals were of analytical reagent (AR) grade and used without any further purification.

### 2.2 Synthesis of pristine and Er-doped LaFeO_3_ nanoparticles

A modified sol–gel approach was employed to synthesize LaFeO_3_ (LFO), La_0.9_Er_0.1_FeO_3_ (LEFO10), La_0.8_Er_0.2_FeO_3_ (LEFO20), and La_0.7_Er_0.3_FeO_3_ (LEFO30). For preparing a 3 g batch, stoichiometric amounts of the precursor salts and citric acid were dissolved in 150 mL of DI water to form a homogeneous solution. Citric acid was added as a chelating agent to bind all the metal cations by forming a complex compound with the metals, maintaining an approximately 1.09 : 1 molar ratio between the total metal cations (La^3+^ + Er^3+^ + Fe^3+^) and citric acid for all compositions. To synthesize LaFeO_3_, an equimolar ratio of lanthanum and iron nitrate was used. The solution was stirred at 400 rpm on a hot plate at a temperature of 80–85 °C for 30 min using a magnetic stirrer. Subsequently, 10 mL of ethylene glycol was added to promote gel stabilization by creating polymeric networks with the metal ions. The solution was then continuously stirred at 400 rpm, maintaining 80–85 °C for approximately 4 h, until a dark-brown, viscous gel was formed. This gel was then dried at 120 °C for about 24 h and ground into fine powder using an agate mortar and pestle. Finally, the resulting powders were annealed at 500 °C for 5 h with a heating rate of 3 °C min^−1^ to obtain the desired nanoparticles.

### 2.3 Material characterizations

X-ray diffraction was utilized for the structural analysis of the synthesized nanoparticles using Cu-Kα radiation of *λ* = 0.154060 nm at 45 kV and 40 mA, and data were collected over a 2*θ* range of 10–80°. The obtained XRD patterns were analyzed by the Rietveld refinement method using HighScore Plus. For the morphological analysis of the samples, a field emission scanning electron microscope (FESEM: JEOL JSM 7600F) was used with an acceleration voltage of 5 kV, and EDS was used to determine the elemental composition. Detailed microstructural examination was carried out using a Transmission Electron Microscope (TEM, Talos F200X), while surface chemical analysis was performed by X-ray Photoelectron Spectroscopy (XPS) using a Thermo Scientific K-Alpha instrument with monochromatic Al Kα radiation.

### 2.4 Preparation of the working electrodes

**Fig. 1:**
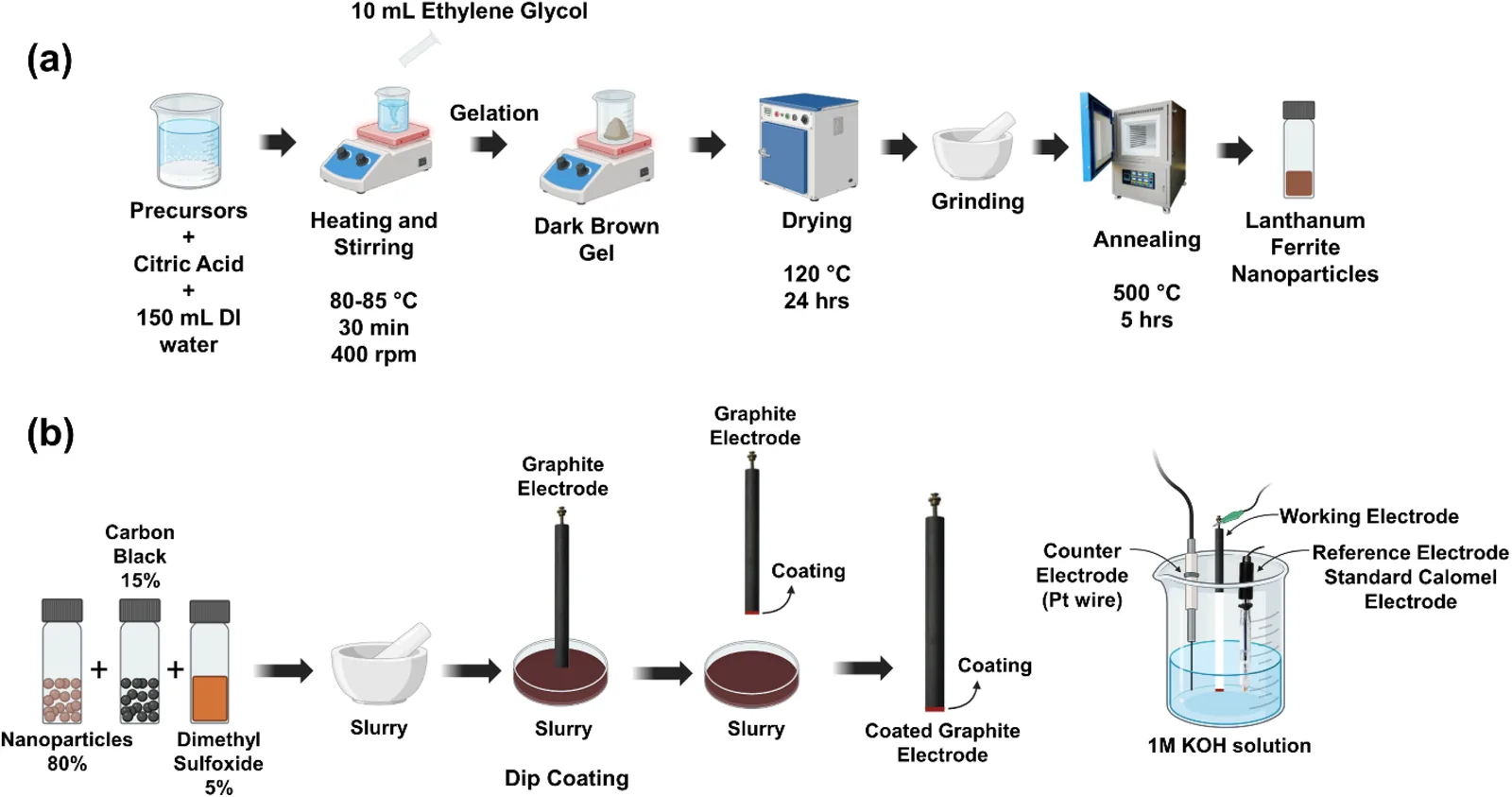
(a) Sol–gel synthesis process of the pristine and Er-doped lanthanum ferrite nanoparticles. (b) Preparation of the working electrodes and the three-electrode electrochemical setup.

The working electrodes were prepared using a slurry of the synthesized nanoparticles, carbon black, and dimethyl sulfoxide, in the mass ratio of 80 : 15 : 5. The slurry was dip-coated onto a conducting graphite rod and dried at 55 °C for a few hours. Dimethyl sulfoxide (DMSO) was used as the dispersing solvent to prepare a homogeneous slurry of the active material prior to coating onto the graphite rod. No additional polymeric binder was employed. After coating, the electrodes were dried to evaporate the solvent, and the resulting oxide layer adhered sufficiently to the graphite substrate, providing adequate mechanical stability for the electrochemical measurements. The mass of the active material was determined from the difference between the weights of the graphite rod before coating and after drying. The average active material loading was approximately 1.5 mg cm^−2^ and was maintained consistently for all electrodes to ensure the reliable comparison of the electrochemical performance. A schematic of the synthesis procedure, working electrode preparation process and electrochemical setup is shown in Fig. 1.

### 2.5 Electrochemical characterization

The electrochemical characterizations, namely, cyclic voltammetry (CV), galvanostatic charge–discharge (GCD), and electrochemical impedance spectroscopy (EIS), of the pristine and Er-doped LaFeO_3_ nanoparticles, LFO, LEFO10, LEFO20, and LEFO30, were performed using a Gamry 3000AE Potentiostat with a conventional three-electrode electrochemical setup to evaluate the intrinsic charge storage behavior of the electrode material. This configuration minimized the influence of device-level factors, thereby enabling a more accurate assessment of the material's fundamental electrochemical properties. It consists of LaFeO_3_-coated electrodes as the working electrode, a standard calomel electrode (SCE) as the reference electrode, and a platinum (Pt) wire as the counter electrode. All three electrodes were immersed in a 1 M KOH solution, which served as the electrolyte. The EIS was carried out using a sinusoidal signal in the frequency range from 0.1 to 10^5^ Hz, CV curves were recorded at different scan rates (5, 10, 20, 50, and 100 mV s^−1^) in the potential window from 0.1 to 0.6 V, and the GCD tests were performed at different current densities (2, 3, 4, and 5 A g^−1^) in the same potential range to probe into the electrochemical properties of the fabricated working electrodes. Each electrochemical measurement was repeated to verify the reproducibility of the observed electrochemical trends. The results presented in this work corresponded to representative measurements obtained under identical experimental conditions. The observed variation between measurements was minimal and did not significantly affect the reported trends.

## 3. Results and discussion

### 3.1 Structural analysis

**Fig. 2:**
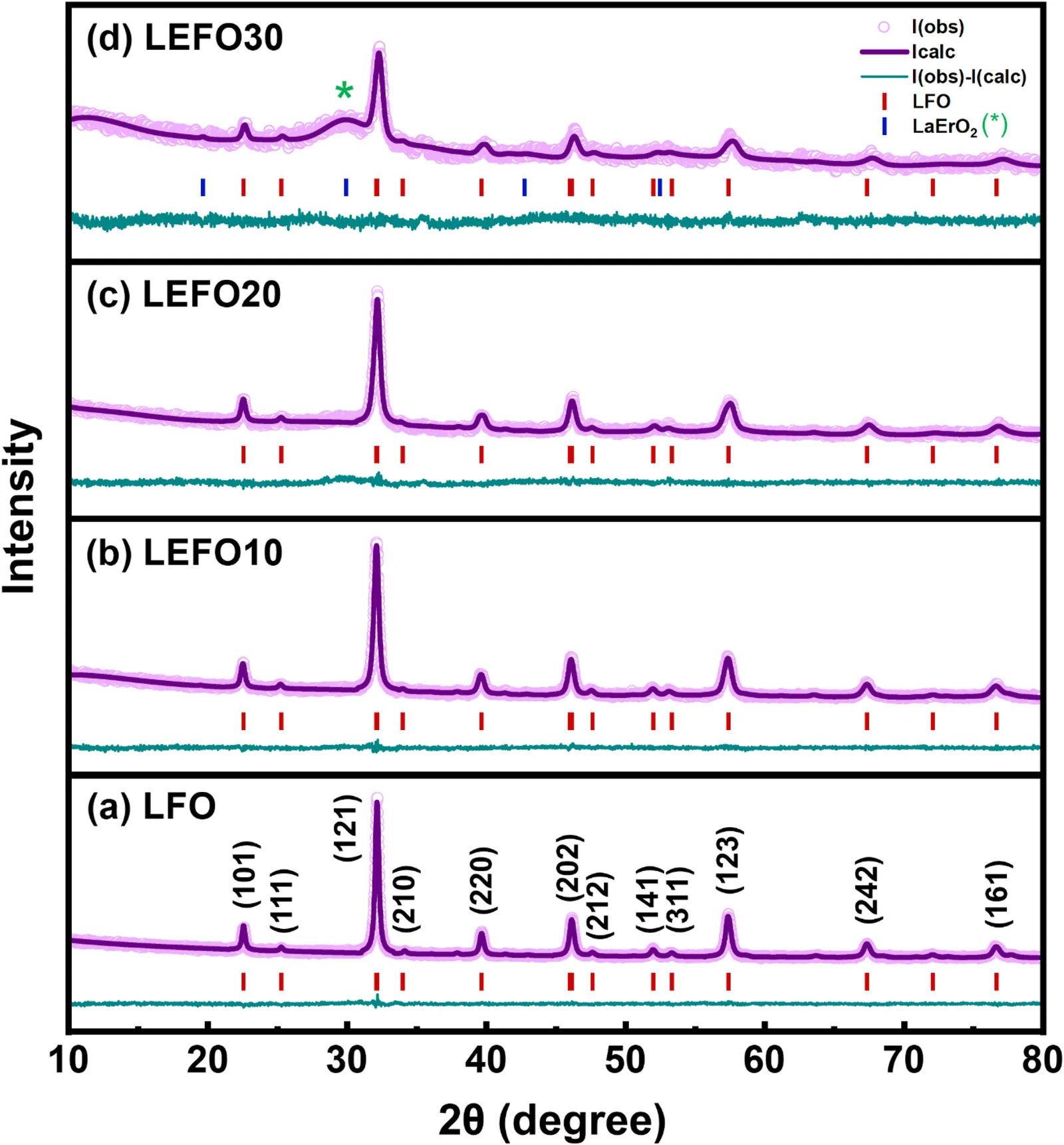
Rietveld analysis of the room-temperature XRD patterns of (a) LFO, (b) LEFO10, (c) LEFO20, and (d) LEFO30.

Fig. 2 shows the room-temperature XRD patterns and the corresponding Rietveld refinements of the pure and Er-substituted LFO nanoparticles. The positions of the peaks (from XRD patterns) and their associated planes match well with the JCPDS card no. 96-152-6451 (LaFeO_3_). The Rietveld refinement was performed with the HighScore Plus software using a pseudo-Voigt peak profile function, and peak broadening was modeled by refining the profile parameters (*U*, *V*, and *W*) and the peak-shape parameters. The close match between the experimental and calculated profiles and the absence of any systematic deviations in the difference plots, as shown in Fig. 2, suggested the reliability of the refinement. As shown in Table 1, the goodness-of-fit (*χ*^2^) values were 1.066, 0.985, 1.462, and 1.047 for LFO, LEFO10, LEFO20, and LEFO30, respectively, indicating reliable refinement with no significant overfitting. Moreover, the refinement also yielded low residual factors (*R*_p_ = 3.816–4.389% and *R*_wp_ = 4.846–5.833%), confirming the quality and reliability of the refinement. As shown in Fig. 2(a), the pure LFO sample sintered at 500 °C for 5 h exhibits a well-defined orthorhombic structure. Er substitution up to 20 mol% maintained a single-phase orthorhombic perovskite structure with space group *Pnma*.^24–26^ The Er-substituted samples exhibited an overall reduction in the unit cell volume, which was attributed to the substitution of large La^3+^ ions with small Er^3+^ ions. The substitution was suggested by the systematic peak shifts of the major peaks toward higher 2*θ* values (summarized in Table S1). No secondary phase was detected up to 20% Er, suggesting the formation of a single-phase solid solution. In contrast, the appearance of a secondary LaErO_2_ phase [Fig. 2(d)] at 30% Er indicated that the solubility limit was exceeded. Quantitative phase analysis from Rietveld refinement revealed approximately 47.7 wt% LaErO_2_ in the LEFO30 sample. Once the limit was reached, excess Er ions could no longer be incorporated into the perovskite lattice and instead formed a separate oxide phase.

**Table 1:** Variation in structural data, reliability factor, crystallite size (D), micro-strain (*ε*), and dislocation density (*δ*) with Er doping in LFO

	Unit cell parameters of the NPs	Rietveld factors	*D* (nm)	*ε* (×10^−3^)	*δ* (×10^−4^) (nm^−2^)
LFO	*a* = 5.57	*R*_p_ = 4.163	33.74	1.76	8.78
	*b* = 7.84	*R*_wp_ = 5.37			
	*c* = 5.55	*R*_exp_ = 5.202			
	*V* = 242.62	*χ* ^2^ = 1.066			
LEFO10	*a* = 5.57	*R*_p_ = 4.028	27.76	2.20	13.45
	*b* = 7.84	*R*_wp_ = 5.112			
	*c* = 5.55	*R*_exp_ = 5.152			
	*V* = 242.46	*χ* ^2^ = 0.985			
LEFO20	*a* = 5.58	*R*_p_ = 4.389	23.40	2.52	18.26
	*b* = 7.84	*R*_wp_ = 5.833			
	*c* = 5.52	*R*_exp_ = 4.825			
	V = 241.36	*χ* ^2^ = 1.462			
LEFO30	*a* = 5.57	*R*_p_ = 3.816	10.69	14	87.51
	*b* = 7.84	*R*_wp_ = 4.846			
	*c* = 5.50	*R*_exp_ = 4.735			
	*V* = 240.21	*χ* ^2^ = 1.047			

The average crystallite size (*D*) was estimated using the Debye–Scherrer equation without correction for instrumental broadening, which may introduce slight overestimation.1
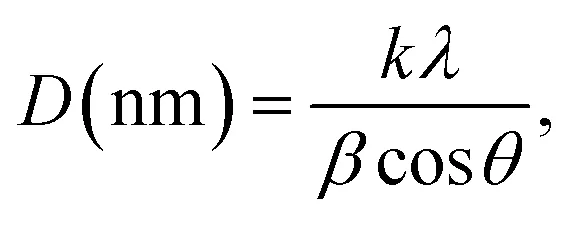
where *k* ∼0.9 is the shape factor, *λ* = 1.5406 Å is the wavelength of Cu Kα_1, 2_ radiation, *β* is the full width at half maximum (FWHM) (in radians) or structural broadening, and *θ* is the Bragg angle (in radians). The variation of structural parameters, refinement factors, crystallite size, microstrain, and dislocation density with Er substitution is summarized in Table 1. The average crystallite size decreased progressively with increasing Er content, consistent with the reduced intensities of the (101), (121), (220), (202), and (123) reflections and the broader diffraction peaks (larger FWHM) compared with those of the undoped LFO shown in Fig. 2. The reduction in crystallite size at higher Er concentrations was attributed to inhibited grain growth arising from the substitution of larger La^3+^ ions by smaller Er^3+^ ions, which introduced lattice strain and suppressed crystal coalescence during sintering.^27,28^

The lattice micro-strain and dislocation density were calculated using the following equations:2
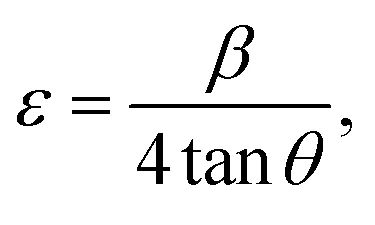
3
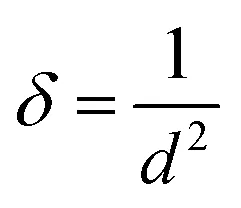


The calculated microstrain values showed a clear increasing trend with high Er content, indicating that Er substitution introduced substantial lattice distortion and internal stress within the crystal framework. The enhanced strain and reduced cell volume collectively suggested that Er doping not only modified the structural symmetry but also induced microstructural refinement and localized lattice disorder in the LFO matrix.^28^ This lattice distortion induced by Er^3+^ substitution modified the defect chemistry of the perovskite lattice, particularly by promoting defect formation and altering Fe valence states, which significantly influenced the electrochemical charge-storage behavior of the material.^29^ However, the excessively high microstrain value obtained for LEFO30 (14 × 10^−3^) could not be attributed solely to substitutional lattice distortion; it resulted from a combined contribution of lattice distortion within the perovskite phase and structural heterogeneity due to the coexistence of secondary phase LaErO_2_.

### 3.2 Morphological analysis

**Fig. 3:**
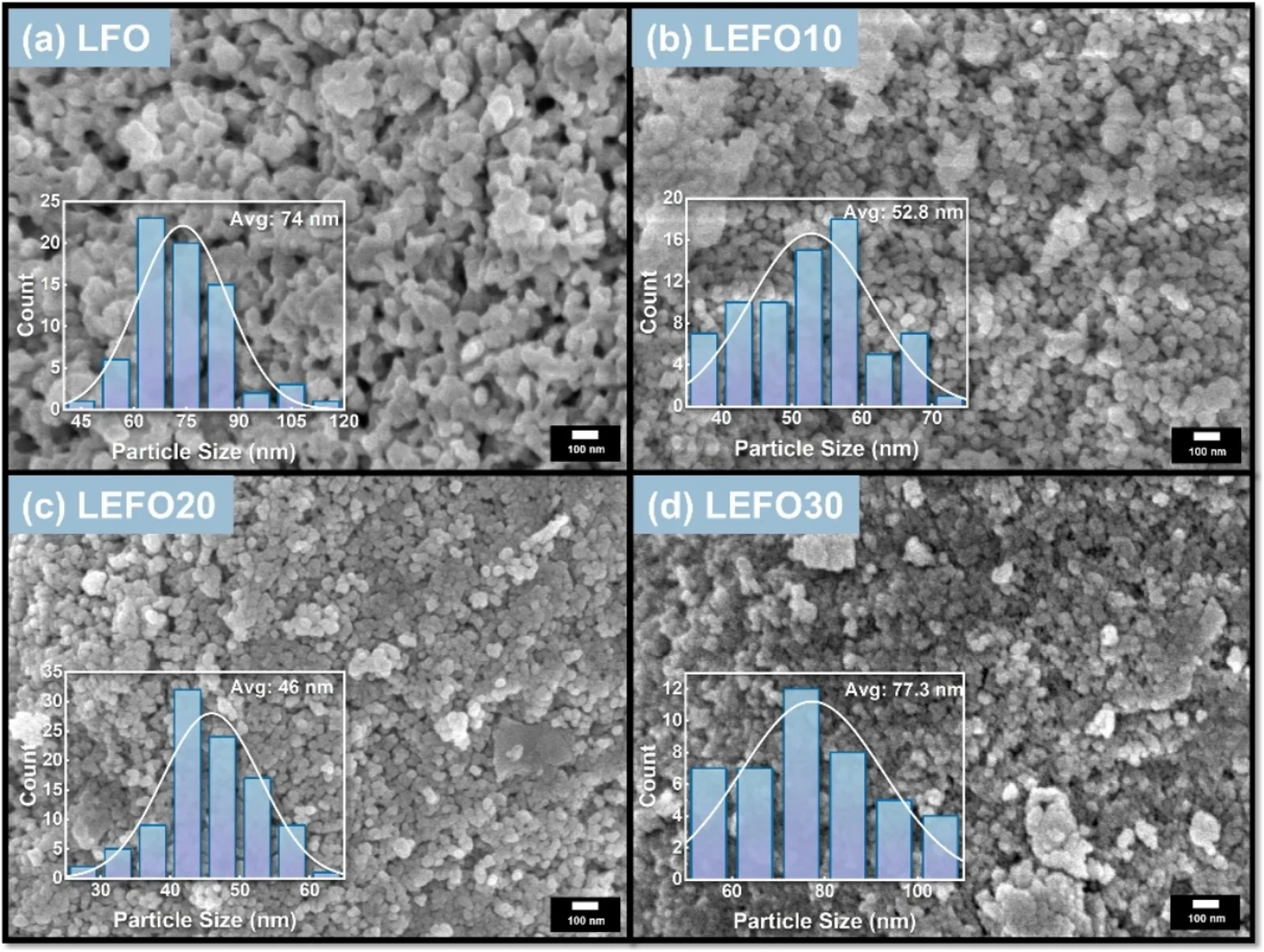
FE-SEM micrographs of pristine and Er-doped LFO (a–d) (Insets: average particle sizes).

Fig. 3(a–d) displays the FE-SEM micrographs of the synthesized nanoparticles, with the corresponding particle size distributions shown in the insets. All samples exhibited agglomerated particle morphologies and a wide range of particle size distributions. Among them, the LEFO30 nanoparticles indicated notably low uniformity, enhanced surface porosity, and pronounced particle agglomeration. These characteristics were likely attributed to particle coalescence and the formation of inhomogeneous secondary impurity phase.^30,31^

The particle sizes of all samples were within the nanometric regime (1–100 nm), suggesting the successful synthesis of nanostructured ferrites. The average particle size decreased from approximately 74 nm for pristine LFO to 46 nm upon 20% Er substitution, indicating that Er incorporation effectively suppressed grain growth up to this doping level. However, at 30% Er substitution (LEFO30), the average particle size increased again to 77.3 nm, suggesting the onset of secondary phase formation and agglomeration at high dopant concentrations. Moreover, the average crystallite sizes estimated from XRD analysis were consistently smaller than the particle sizes observed in FE-SEM images, further supporting the presence of particle agglomeration.^32^

Fig. S(1–4) shows the multi-point EDS analyses of the synthesized nanoparticles, confirming the presence of all expected constituent elements. La, Er, Fe, and O were consistently detected across all compositions, and the corresponding Fe : La ratios are summarized in Table 2. A gradual decrease in the atomic percentage of La with increasing Er doping indicated the progressive substitution of La^3+^ by Er^3+^ ions. The undoped LFO nanoparticles exhibited only La, Fe, and O peaks, with no detectable impurities, whereas the Er-substituted samples displayed additional peaks corresponding to Er, suggesting the successful incorporation of Er^3+^ ions into the ferrite lattice.

**Table 2:** Fe:La atomic ratios

Composition	Fe : La (atomic%)
LFO	1 : 1.06
LEFO10	1 : 1
LEFO20	1 : 0.87
LEFO30	1 : 0.82

**Fig. 4:**
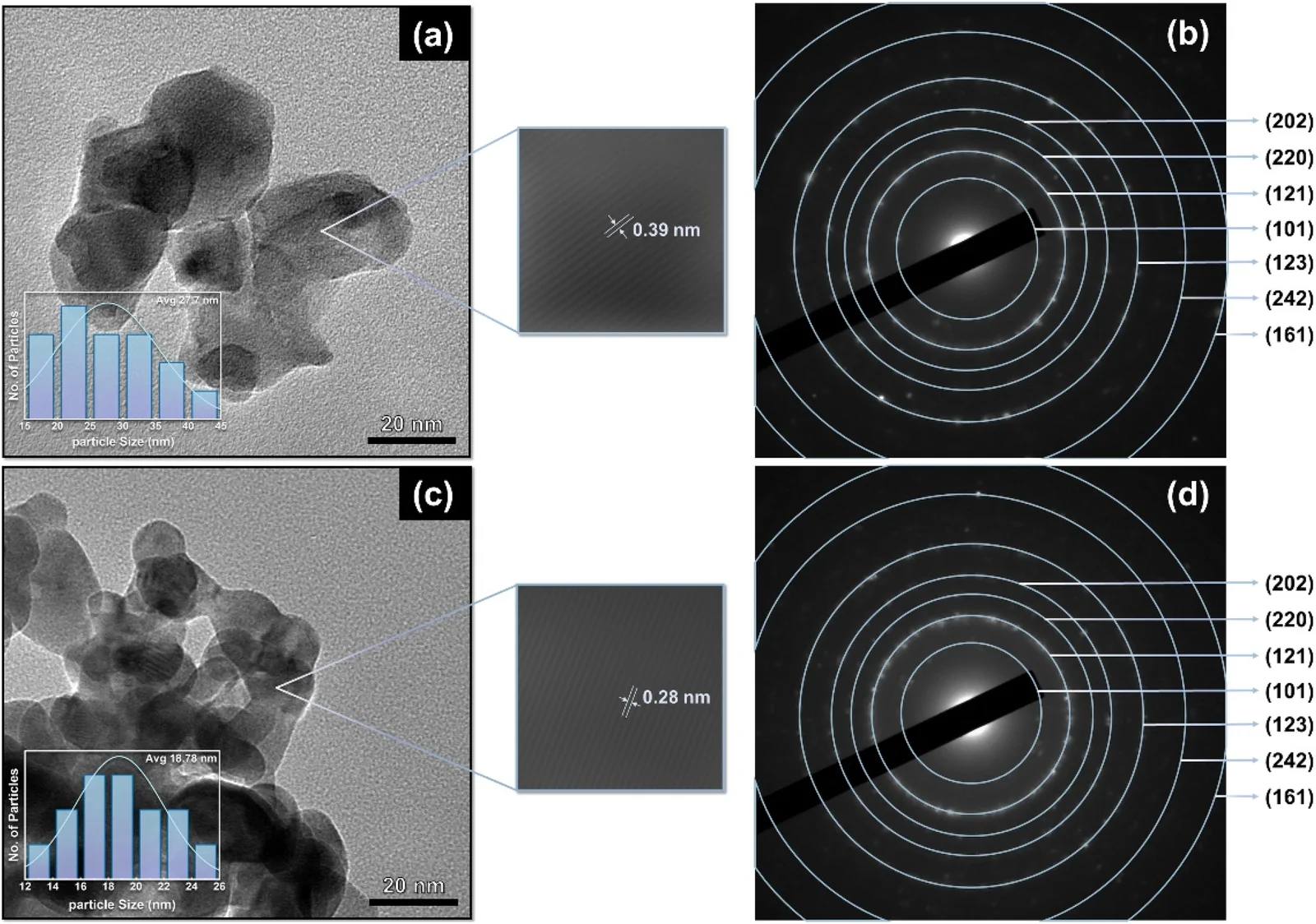
HRTEM images with lattice fringes (insets: particle size distribution histograms) and SAED patterns with indexed planes of (a and b) LFO and (c and d) LEFO20.

High-resolution transmission electron microscopy (HRTEM) was carried out for the pure LFO and LEFO20 samples, the latter exhibiting the highest electrochemical activity and the most prominent lattice strain not associated with secondary phases. Fig. 4(a–d) presents the HRTEM images, particle size distribution histograms, and selected area electron diffraction (SAED) patterns of the LFO and LEFO20 nanoparticles. The SAED patterns [Fig. 4(b) and (d)] showed concentric rings composed of discrete bright spots, suggesting the polycrystalline nature of the samples, which was consistent with the XRD results. The TEM micrographs [Fig. 4(a) and (c)] revealed lattice fringes with multiple orientations, a characteristic feature of polycrystalline materials. Although the SAED patterns suggested the polycrystalline nature of the samples, the HRTEM images showed continuous lattice fringes within individual nanoparticles, indicating that most particles behaved as single-crystalline domains. Consequently, distinct grain boundary or defect contrast was not prominently visible in the recorded HRTEM images.

The HRTEM images showed nearly spherical nanoparticles with minor shape irregularities and a slight tendency toward agglomeration. The particle size distribution histograms [insets of Fig. 4(a) and (c)] indicated average particle sizes of approximately 28 nm for LFO and 19 nm for LEFO20, supporting the grain refinement observed from XRD analysis. The significant size reduction upon Er^3+^ incorporation was attributed to a dopant-induced lattice strain, which hindered particle growth during synthesis. The particle size estimated from HRTEM appeared smaller than that observed in SEM images. This difference arises because SEM primarily reflects the size of aggregated particles, whereas HRTEM enables the visualization of individual primary nanocrystals that are better dispersed during TEM sample preparation.^33,34^

Each diffraction ring in the SAED pattern was indexed to its corresponding (*hkl*) plane by comparing the calculated *d*-spacing values, derived from the ring diameters, with those listed in the Powder Diffraction File (PDF) for LaFeO_3_ (reference code 1 526 450).^35^ The application of the Fast Fourier Transform (FFT) algorithm to the high-resolution TEM images produced distortion-free lattice fringe images [Fig. 4(a) and (c) insets], from which the interplanar spacings were determined. The measured *d*-spacing values, *d*_LFO_ = 0.39 nm and d_LEFO20_ = 0.28 nm, corresponded to the (101) and (121) planes of LFO, respectively, as verified using the reference PDF data. These planes are frequently observed in the orthorhombic perovskite LaFeO_3_ due to their relatively low surface energy and structural stability associated with the FeO_6_ octahedral framework.^36^ The HRTEM observations were consistent with the SAED results, suggesting the crystalline nature of the nanoparticles. In addition, the (121) plane exhibited the highest intensity peak in the XRD patterns, suggesting a preferential orientation of the nanoparticles along this crystallographic direction.

Table 3 compares the *d*-spacing values obtained from the HRTEM lattice fringes with those derived from the XRD-refined lattice parameters for LFO and LEFO20. The close agreement between the measured and calculated values suggested the structural consistency between diffraction and imaging analyses. This coherence strongly supported that the dopant ions were successfully incorporated into the perovskite lattice rather than forming secondary impurity phases.

**Table 3:** Comparison of the interplanar spacings obtained from XRD and HRTEM for LFO and LEFO20

Composition	Plane (*hkl*)	*d* ^calc^_*hkl*_(nm)	*d* ^TEM^_*hkl*_(nm)
LFO	(101)	0.3937	0.39
	(121)	0.2781	—
LEFO20	(101)	0.3935	—
	(121)	0.2780	0.28

These nanoscale structural characteristics revealed by the HRTEM analysis also had important implications for the electrochemical performance of the electrodes. The reduced particle size and improved crystallinity observed for the Er-doped sample increased the electrochemically active surface area and provided shorter ion diffusion pathways, thereby facilitating fast redox reactions and enhanced charge-storage capability. In addition, the presence of well-defined lattice fringes associated with stable crystallographic planes contributed to improved structural stability during repeated charge–discharge processes.^37,38^

### 3.3 XPS analysis

XPS was carried out to examine the elemental composition and chemical states of the LFO and LEFO20 samples. The survey spectra (Fig. S5) suggested the presence of La, Fe, and O in all samples, along with the characteristic Er signal in LEFO20, verifying successful dopant incorporation.

**Fig. 5:**
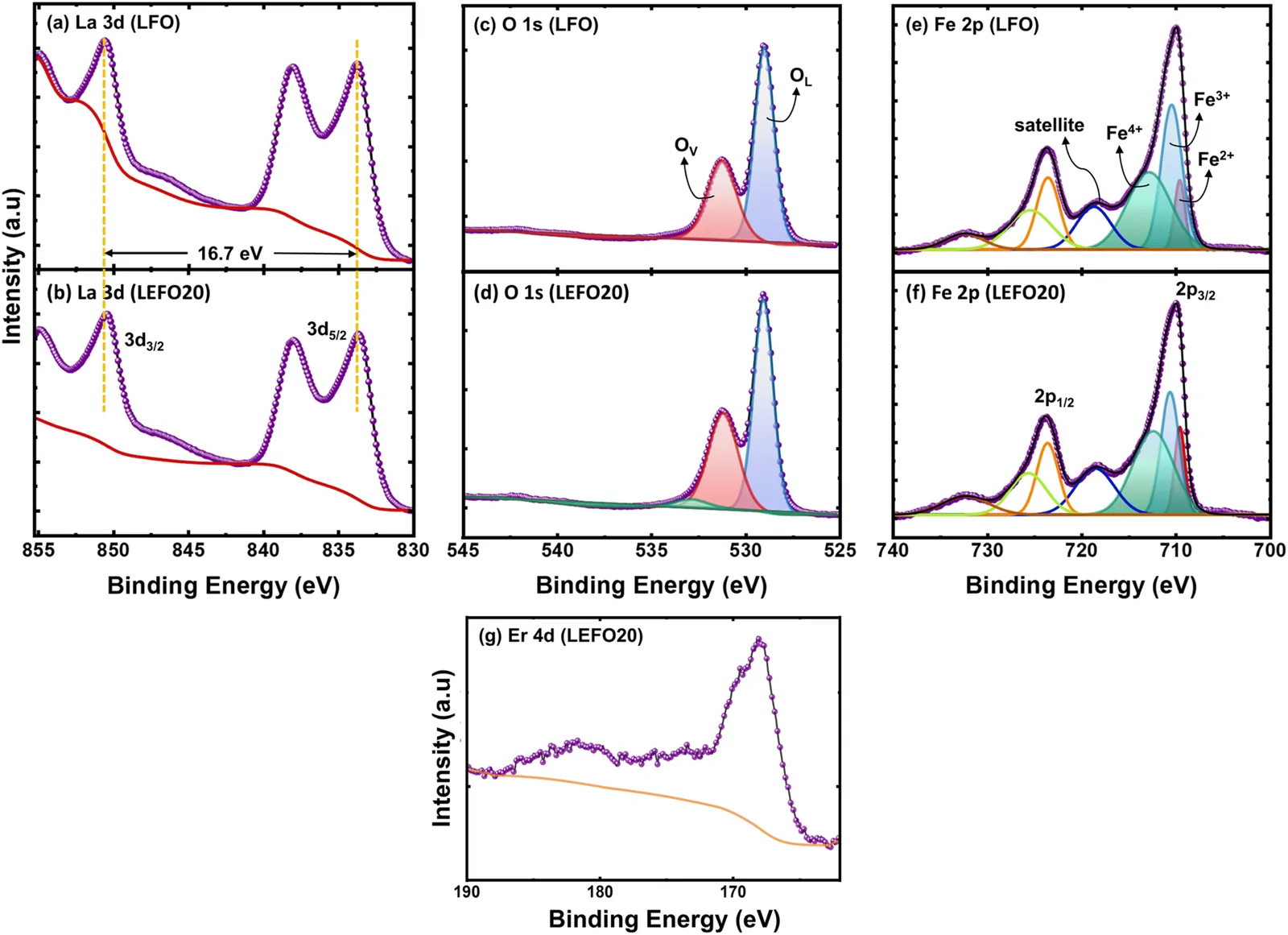
XPS core-level spectra of LFO and LEFO20. (a and b) La 3d spectra showing the 3d_5/2_ and 3d_3/2_ states. (c and d) O 1s spectra with contributions from lattice oxygen (O_L_) and oxygen vacancies (O_V_). (e and f) Fe 2p spectra displaying the Fe^4+^ and Fe^3+^ states. (g) Er 4d spectra.

The high-resolution XPS spectra are presented in Fig. 5. In the La 3d spectra [Fig. 5(a and b)], two pairs of peaks were observed at approximately 833.7 and 850.4 eV, corresponding to the La 3d_5/2_ and La 3d_3/2_ energy levels, respectively. The spin–orbit splitting ΔBE (La 3d_5/2_ – La 3d_3/2_) = 16.7 eV between these levels suggested the presence of La^3+^ ions in oxide form, consistent with previous reports.^39,40^

The O 1s spectra [Fig. 5(c and d)] were deconvoluted into three components. The dominant peak centered at ∼529.1 eV was assigned to the lattice oxygen (O^2−^) in the LaFeO_3_ lattice. The high-binding-energy component near ∼531.3 eV was attributed to the surface oxygen species associated with C–O bonds and/or metal carbonate species, while the component at a higher binding energy (∼532–533 eV) was assigned to the organic C=O species adsorbed on the sample surface.^41^ These oxygen-containing species commonly arise from atmospheric exposure during sample handling and are frequently observed in oxide materials.^42,43^ Accordingly, the O 1s spectra were used to identify the chemical environments of oxygen but were not employed to quantify oxygen vacancy concentration.^43^

Fig. 5(e and f) shows the high-resolution Fe 2p spectra of LFO and LEFO20 recorded at room temperature. The Fe 2p spin–orbit components split into Fe 2p_3/2_ and Fe 2p_1/2_ states, corresponding to multiple iron oxidation states. In the Fe 2p_3/2_ region, peaks at 709.08, 710.48, and 712.88 eV were attributed to the Fe^2+^, Fe^3+^, and Fe^4+^ species in the Fe 2p_3/2_ region, respectively.^44^ Quantitative peak-area analysis afforded Fe^2+^: (Fe^2+^ + Fe^3+^ + Fe^4+^) ratios of 0.101 for LFO and 0.138 for LEFO20, indicating an increased Fe^2+^ concentration upon Er^3+^ substitution. This increase suggested that Er incorporation modified the local electronic structure and promoted defect-mediated charge compensation within the LFO lattice. Owing to the smaller ionic radius of Er^3+^ compared with that of La^3+^, A-site substitution introduced lattice distortion, which altered the Fe–O bonding environment and facilitated the partial reduction of Fe^3+^ to Fe^2+^.^45,46^ Likewise, the Fe^4+^: (Fe^2+^ + Fe^3+^ + Fe^4+^) ratios increased from 0.473 for LFO to 0.520 for LEFO20, demonstrating the enrichment of Fe^4+^ with Er incorporation. The formation of Fe^4+^ species was attributed to charge compensation associated with A-site substitution, where the lattice accommodated the local structural distortion through the partial oxidation of Fe^3+^ to Fe^4+^.^47–49^ The simultaneous increase in Fe^2+^ and Fe^4+^ concentrations indicated that Er substitution modified the defect chemistry and electronic structure of LaFeO_3_ rather than causing surface reduction during XPS measurements. These changes, together with the lattice distortion introduced by Er substitution, facilitate defect-assisted charge transport, reduce the charge-transfer resistance, and enhance ionic diffusion during electrochemical cycling, thereby contributing to the improved electrochemical performance of LEFO20.^46,50^

The modification of the Fe valence distribution, together with the lattice distortion induced by Er substitution, enhanced defect-mediated charge transport, reduced the charge-transfer resistance, and facilitated ionic diffusion during electrochemical cycling, thereby contributing to the improved electrochemical performance of LEFO20.^51^

Fig. 5(g) shows the Er 4d XPS spectra of LEFO20, with one peak located at approximately 168.18 eV, suggesting the presence of Er^3+^ ions. No evidence of erbium clustering or secondary oxide phases was detected in the XRD or TEM analyses, supporting the successful incorporation of Er^3+^ into the LFO lattice. Although XPS is a surface-sensitive technique, the consistency between XPS, XRD, and EDS results suggests that the observed chemical states are representative of the overall material composition.

### 3.4 Electrochemical performance analysis

The electrochemical performance of the synthesized nanoparticles, LFO, LEFO10, LEFO20, and LEFO30, was investigated by performing CV, GCD, and EIS measurements utilizing an electrochemical cell consisting of a three-electrode system (Fig. 1).

#### 3.4.1 Electrochemical impedance spectroscopy (EIS)

**Fig. 6:**
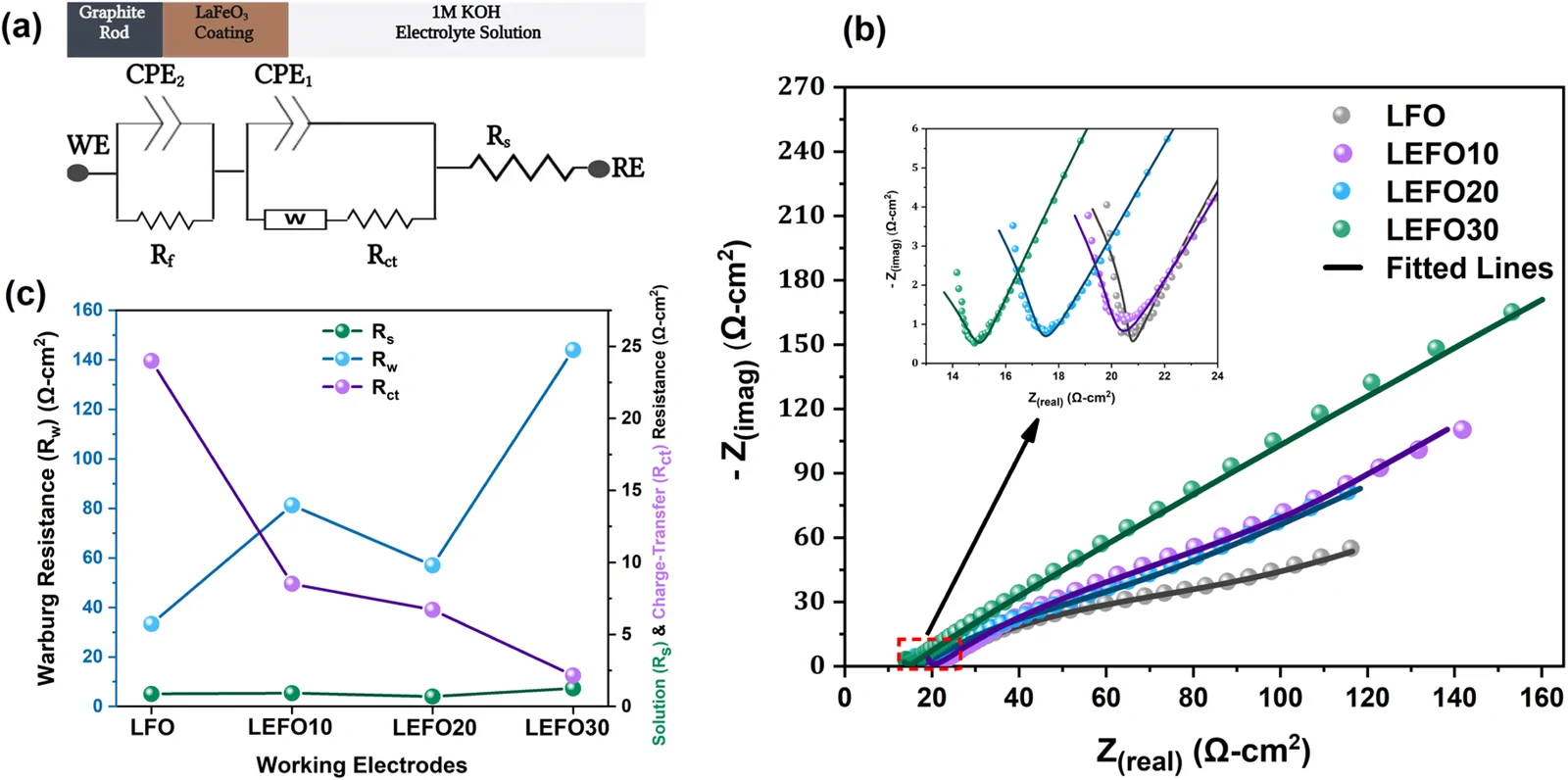
(a) Equivalent electrical circuit (EEC), (b) Nyquist plots of the obtained impedimetric data, and (c) comparison of the Warburg resistance (*R*_w_), solution resistance (*R*_s_), and charge transfer resistance (*R*_ct_).

Electrochemical impedance spectroscopy (EIS) was performed to analyze the ion diffusion tendency and the resistive properties of the working electrodes at the coating/electrolyte interface and in the bulk of the coating. The EIS technique probes into an electrochemical system to characterize and differentiate between various electrical, electrochemical, and physical processes taking place.^52^ A typical electrochemical system comprises several components, *e.g.*, solution resistance, charge-transfer resistance, double-layer capacitance, and bulk resistance, which can be modelled using an equivalent electrical circuit (EEC) consisting of elements, such as resistors, capacitors, constant phase elements (CPE), and a Warburg element. Before modelling an EEC, the obtained data must be validated by the Kramers–Kronig test.^52^ The Kramers–Kronig test was performed using the EChem Analyst software, and the fractional residual errors for the real and imaginary impedances are plotted in Fig. S6. The error% for all the electrodes was less than 0.5%, which confirmed the linearity, causality, stability, and finiteness of the electrochemical system.^52^ After the validation of the impedimetric data using the Kramers–Kronig test, they were modelled using the EEC,^53–55^ as shown in Fig. 6(a), and the fitted Nyquist plots are shown in Fig. 6(b). The EEC consisted of the solution resistance (*R*_s_), the charge transfer resistance (*R*_ct_) at the coated film/electrolyte interface, the Warburg component (*W*) as the impedance for ions to diffuse into the coating, and the bulk resistance (*R*_f_) of the coated film. Besides, the Nyquist plots represent the real impedance (resistance) on the *x*-axis and the imaginary impedance (reactance) on the *y*-axis. On the Nyquist plot, the low-frequency region corresponds to high impedance values characterized by an inclined straight line. In contrast, the high-frequency region corresponds to low impedance values characterized by a semicircle.^52^ But from the Nyquist plots depicted in Fig. 6(b), the semicircular region is not well-defined, suggesting a higher Warburg resistance than the charge-transfer resistance^52^ (which complies with the values of *R*_w_ and *R*_ct_ presented in Table 4). For such instances, the graphical analysis to identify the *R*_ct_ (diameter of the semicircle) would not be reliable. So, the EEC fitting was performed using the EChem Analyst software, and the fitted parameters are listed in Table 4. The reliability of the fitted impedance parameters was supported by the excellent agreement between the experimental and simulated Nyquist plots, along with the low residual error obtained from the Kramers–Kronig validation. These results suggested that the selected equivalent circuit model appropriately represented the electrochemical processes occurring at the electrode/electrolyte interface.

**Table 4:** Fitted parameters of the equivalent electrical circuit (EEC) and their goodness of fit

	Solution resistance (*R*_s_) (Ω cm^2^)	Bulk resistance (*R*_f_) (Ω cm^2^)	Charge transfer resistance (*R*_ct_) (Ω cm^2^)	Warburg resistance (*R*_w_) (at 0.1 Hz) (Ω cm^2^)	Goodness of fit (×10^−6^)
LFO	5.08	6.62	23.99	33.34	82.82
LEFO10	5.40	3.61	8.51	81.3	470.6
LEFO20	4.03	4.11	6.71	57.0	92.27
LEFO30	7.34	8.95	2.15	144	218.7

The values of the solution resistances (*R*_s_) plotted in Fig. 6(c) were in the range of 4–7 Ω cm^2^, suggesting similar electrolyte conditions for all the working electrodes. Besides, the bulk resistance (*R*_f_-resistance of the film coated on the graphite rod) of the LEFO10 electrode was 3.61 Ω cm^2^. However, upon increasing the doping concentration, it increased to 4.03 Ω cm^2^ for LEFO20 and reached 7.34 Ω cm^2^ for LEFO30. With the increase in Er concentration, more La atoms were replaced by Er atoms. Due to the electronic configuration of La^3+^ ([Xe] 4f^0^) and Er^3+^ ([Xe] 4f^11^), there were fewer vacant orbitals for Er^3+^ in comparison to La^3+^, which resulted in lower conductivity with an increase in dopant concentration.^56^ Though the *R*_f_ value of the LFO electrode showed anomalous behavior, the other three electrodes complied with the decrease in conductivity with the increase in Er concentration. Again, the Warburg impedance parameter expresses the difficulty of electrolyte ions to diffuse into the film coated on the working electrode through the film/electrolyte surface. On the other hand, the charge transfer resistance is the resistance for the redox reaction to occur at the electrode/electrolyte interface. The Warburg resistance (*R*_w_), the real part of *Z*_w,_ is calculated using eqn (S1) and tabulated in Table 4. The highest Warburg resistance (*R*_w_) for LEFO30 was due to the formation of the secondary phase (lanthanum erbium oxide), which blocked the diffusion pathways for oxygen anion intercalation [charge storage mechanism-2]. On the other hand, the lowest Warburg resistance (*R*_w_) of LEFO20 was attributed to the increase in defect concentration due to doping compared to LFO (as suggested by the XPS analysis). A quantitative analysis of the impedance parameters revealed that the electrochemical performance was strongly governed by ion diffusion and charge-transfer processes. Although the Warburg resistance (*R*_w_) and charge-transfer resistance (*R*_ct_) provided valuable information regarding ion diffusion and interfacial redox kinetics, neither parameter alone determined the overall electrochemical performance. The superior capacitive behavior of LEFO20 arose from the synergistic combination of enhanced defect concentration, reduced particle size, preserved single-phase perovskite structure, and sufficiently low ion-diffusion and charge-transfer resistances. In contrast, although LEFO30 exhibited comparatively favorable charge-transfer characteristics, the formation of the secondary LaErO_2_ phase and the accompanying increase in particle size reduced the number of electrochemically active sites and obstructed oxygen-ion diffusion, leading to inferior electrochemical performance. These results indicated that the optimum performance of LEFO20 was achieved through a balanced contribution of ion diffusion, charge-transfer kinetics, defect chemistry, and electrochemically active surface area, rather than by minimizing a single impedance parameter. In particular, the lower Warburg resistance (*R*_w_) observed for the LEFO20 electrode indicated enhanced ion diffusion within the electrode matrix, which could be attributed to the Er-induced lattice distortion and defect-mediated electronic structure modification, as supported by the XRD and XPS results. Similarly, variations in charge-transfer resistance (*R*_ct_) reflected differences in interfacial redox kinetics, while the bulk resistance (*R*_f_) was associated with the intrinsic electronic conductivity of the electrode material. The combined reduction in *R*_w_ and *R*_ct_ for the optimally doped composition facilitated improved electrochemical activity. Therefore, although moderate Er substitution improved defect-assisted electrochemical transport, excessive Er incorporation, resulting in LaErO_2_ phase segregation, became detrimental to electrochemical performance due to the obstruction of ion diffusion pathways and the degradation of charge-transfer processes.

#### 3.4.2 Cyclic voltammetry (CV)

**Fig. 7:**
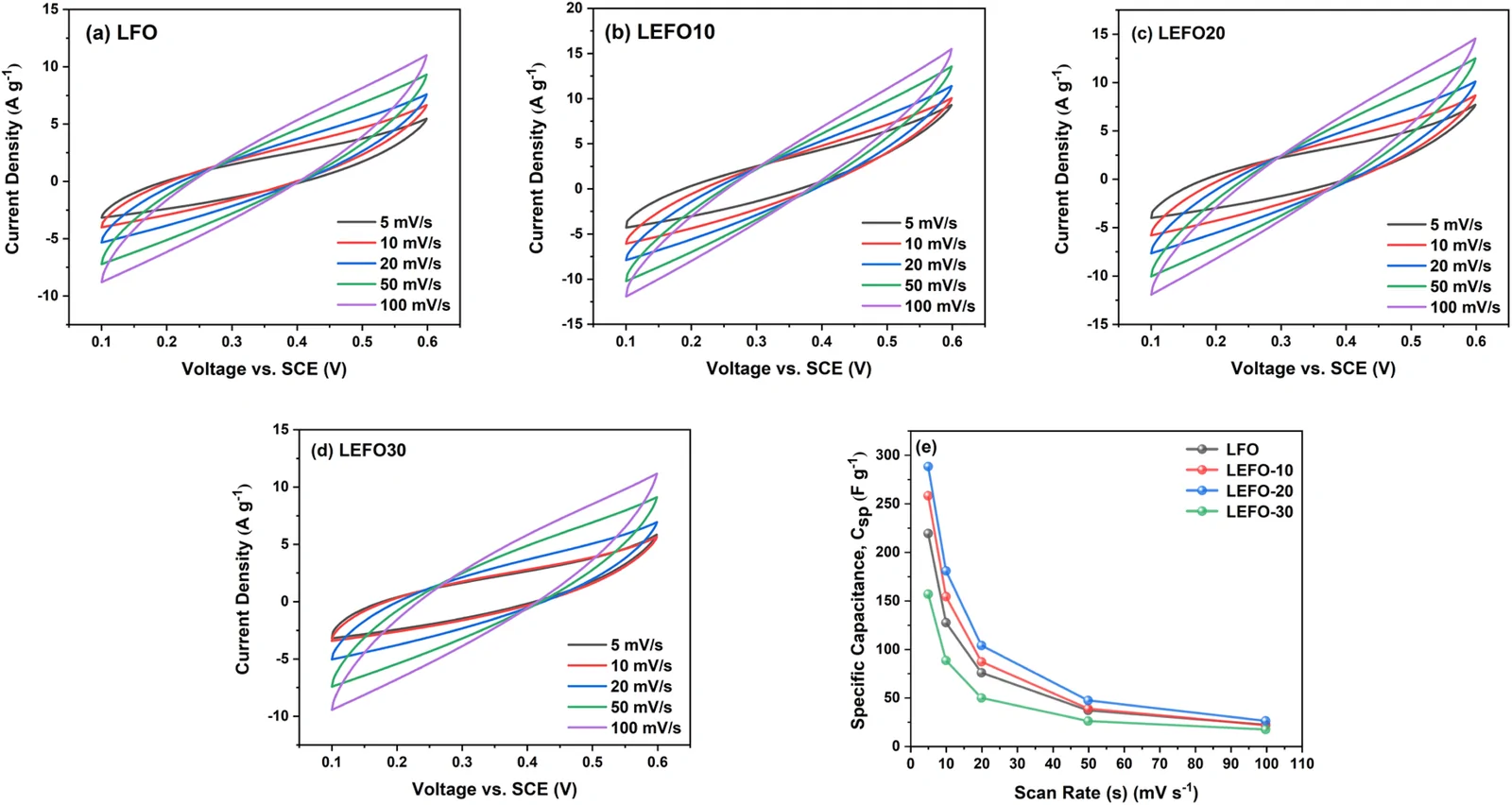
Cyclic voltammograms of the working electrodes: (a) LFO, (b) LEFO10, (c) LEFO20, and (d) LEFO30, at different scan rates. (e) Plot of *C*_sp_*vs.* scan rate.

Cyclic voltammetry (CV) was used to study the charge-transfer rate at the electrode/electrolyte interface. Rectangular cyclic voltammograms are indicative of the electrical double-layer capacitor (EDLC) type of supercapacitors,^57^ whereas any deviation from the rectangular shape suggests the pseudocapacitive behavior of supercapacitors.^58^ The cyclic voltammetry curves for LFO, LEFO10, LEFO20, and LEFO30, as shown in Fig. 7(a)–(d), exhibited quasi-rectangular shapes, indicating mixed EDLC and pseudocapacitive behavior.^57–59^ The EDLC behavior of the electrode materials was due to the electrostatic charge adsorption at the interface of the electrode/electrolyte, involving no charge transfer between the electrolyte and the electrode material.^60^ On the other hand, the pseudocapacitive behavior was due to the faradaic surface redox reactions (known as Redox Pseudocapacitance) or the intercalation and deintercalation of ions (known as Intercalation Pseudocapacitance) in the bulk of the material.^60,61^

**Fig. 8:**
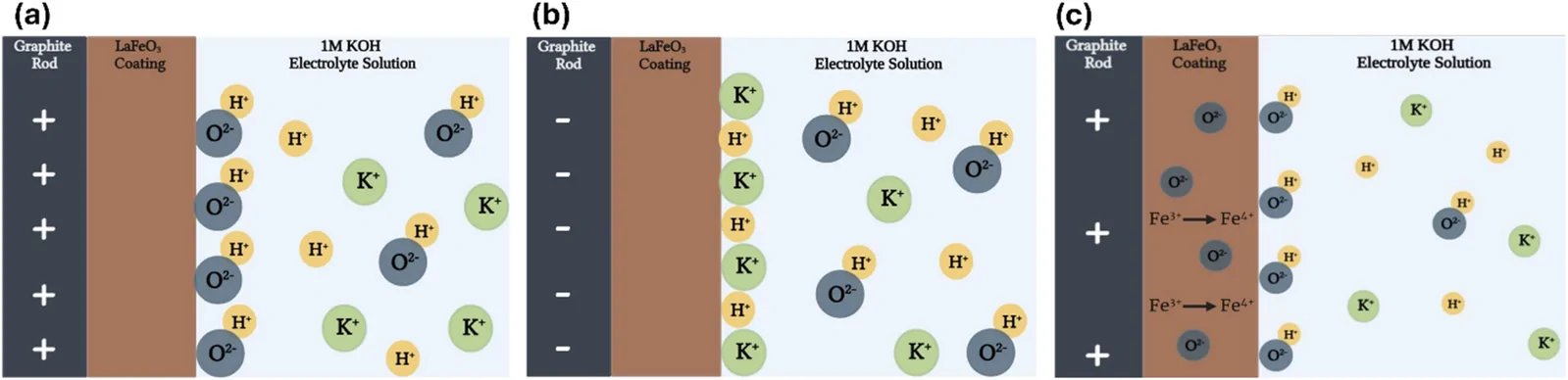
Charge storage mechanisms: (a) adsorption during the negative bias at the electrode, (b) adsorption during the positive bias at the electrode, and (c) anion-intercalation mechanism.

**Fig. 9:**
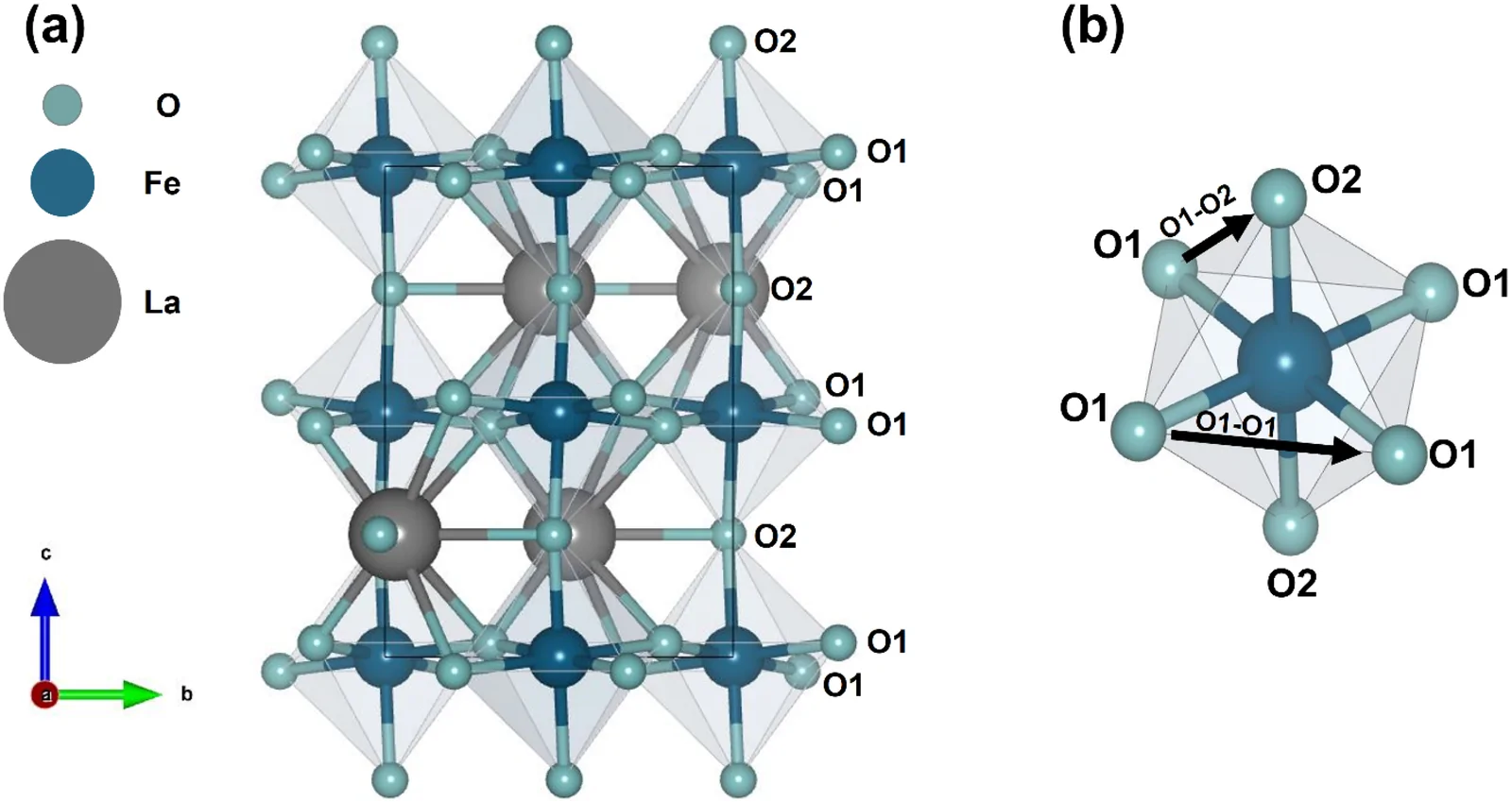
(a) Perovskite structure of LaFeO_3_, (b) FeO_6_ octahedron and the O1–O1 and O1–O2 diffusion pathways.

From the shape of the cyclic voltammograms and the inherent properties of the perovskite oxides, which exhibited high oxygen vacancies,^61^ strong oxygen adsorption ability,^9^ high oxygen ion conductivity, and better surface oxygen exchange kinetics,^8^ two charge-storage mechanisms were proposed to explain the increase in the values of specific capacitance (*C*_sp_):

(1). Charges are stored at the electrode/electrolyte interface due to the electrostatic attraction of the electrode bias towards the oppositely charged ions present in the electrolyte solution. The absorption and desorption of ions during the forward and reverse potential sweeps are demonstrated in Fig. 8(a) and (b). These processes can be expressed by the following reactions:^4^(LaFeO_3_)_surface_ + OH^−^ ↔ [(LaFeO_3_)^+^OH^−^]_surface_ + e^−^,(LaFeO_3_)_surface_ + K^+^ + e^−^ ↔ [(LaFeO_3_)^−^K^+^]_surface_,(LaFeO_3_)_surface_ + H^+^ + e^−^ ↔ [(LaFeO_3_)^−^H^+^]_surface_.

(2). Charges can be stored in the bulk of the material due to the oxygen anion intercalation (Fig. 8(c)) at the oxygen vacancy sites present in the perovskite oxide (LaFeO_3_). In sub-stoichiometric perovskites, OH^−^ ions from the 1 M KOH electrolyte adsorb on the electrode surface and generate O^−^ species *via* proton transfer, which intercalate into oxygen vacancies and migrate along the octahedral edges of FeO_6_ (Fig. 9(b)), causing the oxidation of the B-site element (Fe^3+^ to Fe^4+^) without any structural change.^4,62^ In LaFeO_3_, ion transport predominantly occurs through the low-energy O1–O1 and O1–O2 pathways between inequivalent oxygen sites (8d and 4c), as the O2–O2 route is energetically unfavorable.^63^ Consequently, this process is governed by both Warburg resistance, *R*_w_ (difficulty of ion diffusion), and the charge transfer resistance, *R*_ct_. The oxygen ion intercalation mechanism can be expressed by the following reaction:^4,61^LaFeO_3−*x*_ + 2α H^−^ ↔ La[Fe_2α_^4+^, Fe_1−2α_^3+^]O_3_ + 2α e^−^ + α H_2_O.

From the cyclic voltammetry curves, the specific capacitance (C_sp_) values were calculated using eqn (4), and they are tabulated in Table 6.4
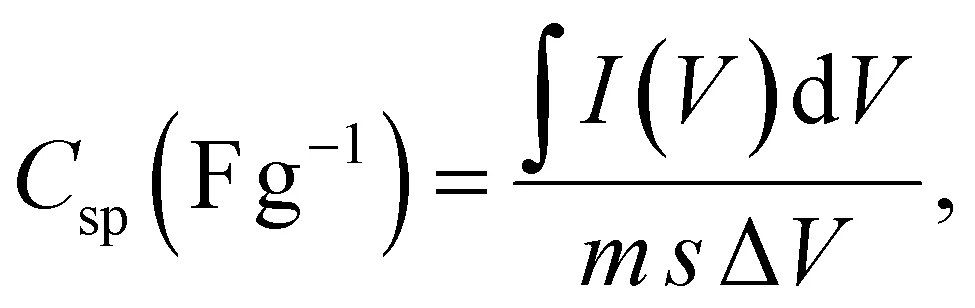
where *C*_sp_ is the specific capacitance, ∫*I*(*V*)d*V* is the integral area of the cyclic voltammograms, ‘*m*’ is the mass of the active electrode material, ‘*s*’ is the scan rate in mV s^−1^, and Δ*V* is the potential window. From eqn (4), the specific capacitance is directly proportional to the integral area under the CV curve. The area of the CV curves increased up to a doping concentration of 20% Er (LEFO20), which caused the C_sp_ value of LFO to increase from 219.07 F g^−1^ to 258.08 F g^−1^ for LEFO10 and 288.22 F g^−1^ for LEFO20 at a potential sweep rate of 5 mV s^−1^. The increase in *C*_sp_ values could be explained by the increase in surface adsorption due to the higher specific surface area, owing to the decrease in particle size, obtained from SEM images (Fig. 3), and the increase in defect concentration in the perovskite oxides due to doping at the A-site, indicated by the XPS analysis. But upon further increment of the doping concentration to 30%, the *C*_sp_ value decreased to 156.43 F g^−1^, which could be attributed to the increase in particle size,^61^ obtained from the SEM images (Fig. 3), and the formation of the secondary phase (lanthanum erbium oxide), identified from the XRD pattern of LEFO30. The increase in particle size reduced the active surface area for oxygen exchange kinetics,^8^ and the introduction of a secondary phase induced hindrance to the diffusion of the ions, causing lower intercalation of the oxygen anions (indicated by the highest Warburg resistance in Table 4). It was worth noting that the enhanced electrochemical performance of the LEFO20 electrode was attributed to substitution-induced defect engineering within the LaFeO_3_ lattice rather than to the electrochemical contribution of an independent Er-containing oxide phase. This interpretation was supported by the structural characterization, which suggested that LEFO10 and LEFO20 retained the single-phase orthorhombic perovskite structure, whereas a secondary lanthanum erbium oxide phase emerged only in LEFO30. Notably, the appearance of this secondary phase was accompanied by a significant decline in the specific capacitance, indicating that the improved charge-storage behavior arose primarily from controlled Er^3+^ substitution and the associated modification of the perovskite defect chemistry, rather than from the formation of an Er-containing secondary phase.

To identify which one of the two proposed mechanisms was dominant, Dunn's method was used, and the obtained capacitive and diffusion-controlled contributions are listed in Table S2. From the values presented in Table S2 (diffusion-controlled contribution is 86.30%, 86.03%, 84.96%, and 89.51% for LFO, LEFO10, LEFO20, and LEFO30, respectively), it is evident that the intercalation mechanism is the dominant charge-storing mechanism. With the increase in doping concentration up to 20 mol%, the capacitive (surface-controlled) contribution increased, which was supported by the decrease in particle size, leading to an increased electrochemically active surface area. The lower capacitive contribution of the LEFO30 electrode was consistent with the increase in particle size obtained from SEM. Again, with increasing scan rate, the capacitive contribution increased, reaching 41.53%, 42.06%, 44.18%, and 34.39% at 100 mV s^−1^ for LFO, LEFO10, LEFO20, and LEFO30, respectively. This trend indicated the coexistence of both diffusion-controlled and surface-controlled processes, with diffusion-controlled intercalation remaining dominant.

As shown in Fig. 7(a)–(d), the maximum current flowing through the working electrode of a specific composition increases with the increase in scan rate 
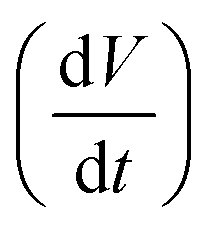
, which can be explained by eqn (5). In addition, for any specific composition, with the decrease in scan rate, the *C*_sp_ value increased significantly (Fig. 7(e)), as the ions got more time to intercalate into the active material's vacant sites (according to Charge-Storage Mechanism-2), thereby enhancing its charge storage capacity.5
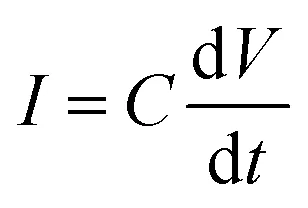


**Fig. 10:**
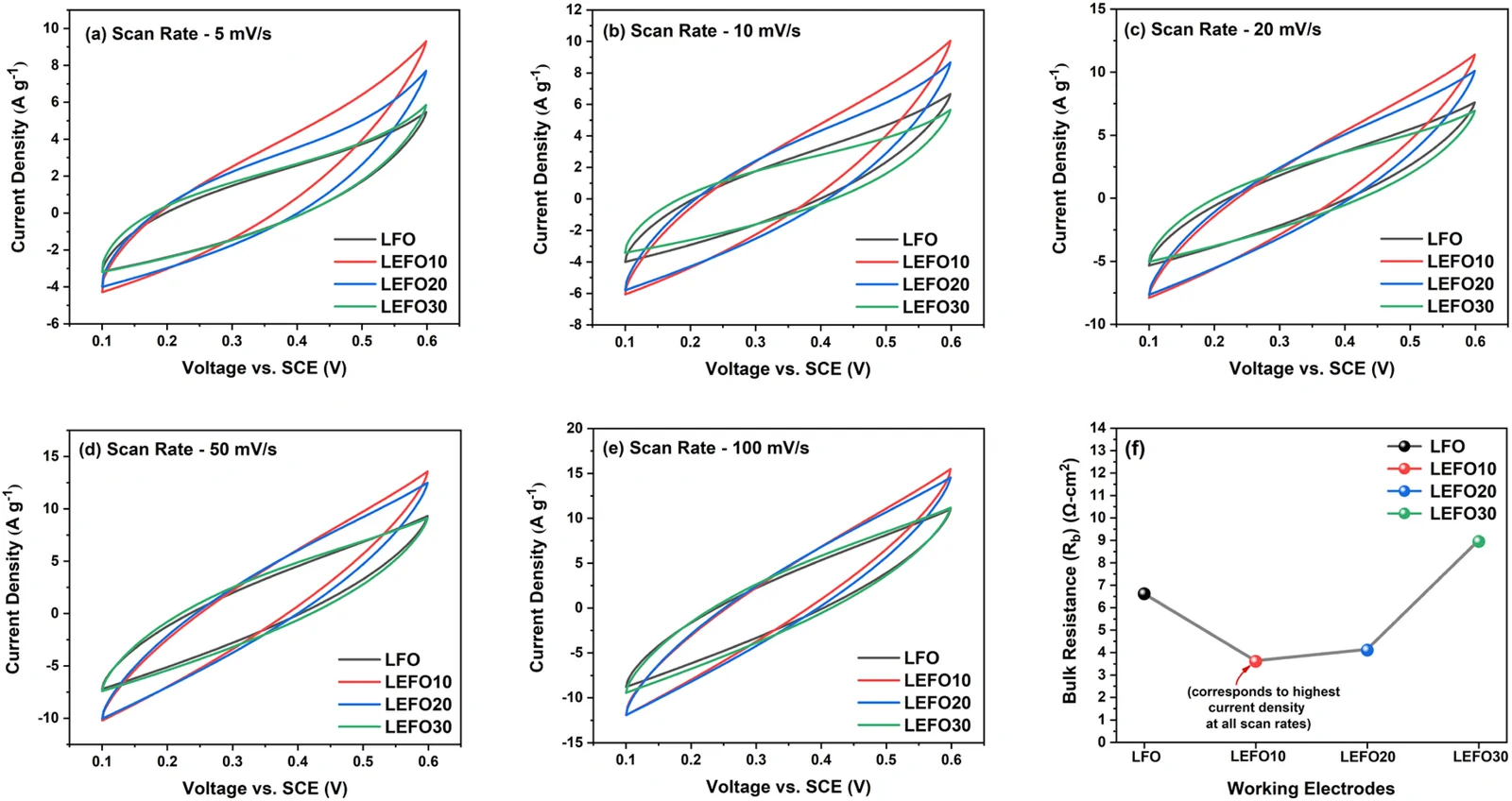
Cyclic voltammograms of LFO, LEFO10, LEFO20, and LEFO30 at (a) 5 mV s^−1^, (b) 10 mV s^−1^, (c) 20 mV s^−1^, (d) 50 mV s^−1^, and (e) 100 mV s^−1^. (f) Bulk resistance (*R*_f_) of the working electrodes.

The CV curves for the electrode materials at definite scan rates, as shown in Fig. 10(a)–(e), highlighted that the highest current density was obtained for the LEFO10 electrode, whereas the current densities of the other electrodes, LEFO20, LFO, and LEFO30, were in descending order. Despite having a higher *C*_sp_ value, LEFO20 had a lower current density compared to LEFO10. The reason behind it was the higher bulk resistance (*R*_f_) of LEFO20 compared to LEFO10 (Table 4), whereas the charge-storing capacity was due to the ease of intercalation of ions (indicated as the Warburg Resistance, *R*_w_) and the faradaic redox reaction (expressed as the charge-transfer resistance, *R*_ct_). The decrease in the diffusion pathway (indicated by the decrease in the bond lengths of O1–O1 and O1–O2, as shown in Table 5) along the edges of the FeO_6_ octahedron in the perovskite oxide lowered the *R*_w_ and *R*_ct_ values, making the diffusion of anions into the defect sites easier^63^ and enhancing the charge storage capacity (Table 6).

**Table 5:** Atomic co-ordinates of the atoms inside perovskite oxide and the octahedral edge lengths

		Atomic co-ordinates			Wyckoff position	Bond length (Å)	
		*x*	*y*	*z*		O1–O1	O1–O2
LFO	La	0.47294	0.25	0.00893	4c	2.8415	2.9536
	Fe	0.0	0.0	0.0	4a		
	O1	0.21657	0.01593	0.30457	8d		
	O2	0.49494	0.25	0.57484	4c		
LEFO10	La	0.46927	0.25	0.00424	4c	2.7987	2.8256
	Fe	0.0	0.0	0.0	4a		
	O1	0.18492	0.02411	0.27611	8d		
	O2	0.51124	0.25	0.58	4c		
LEFO20	La	0.46620	0.25	0.002585	4c	2.7975	2.4838
	Fe	0.0	0.0	0.0	4a		
	O1	0.17872	0.04871	0.23063	8d		
	O2	0.52467	0.25	0.58012	4c		
LEFO30	La	0.46301	0.25	0.007	4c	2.8049	3.0190
	Fe	0.0	0.0	0.0	4a		
	O1	0.174628	0.01434	0.28063	8d		
	O2	0.408359	0.25	0.55952	4c		

**Table 6:** Specific capacitances (*C*_sp_) of the working electrodes at different scan rates

Scan rate (mV s^−1^)	Specific capacitance, *C*_sp_ (F g^−1^)			
	LFO	LEFO10	LEFO20	LEFO30
5	219.07	258.08	288.22	156.43
10	126.97	153.81	180.42	87.92
20	75.21	86.55	103.37	49.24
50	36.34	38.21	46.61	25.30
100	21.46	20.95	25.48	16.59

#### 3.4.3 Galvanostatic charge–discharge (GCD) analysis

**Fig. 11:**
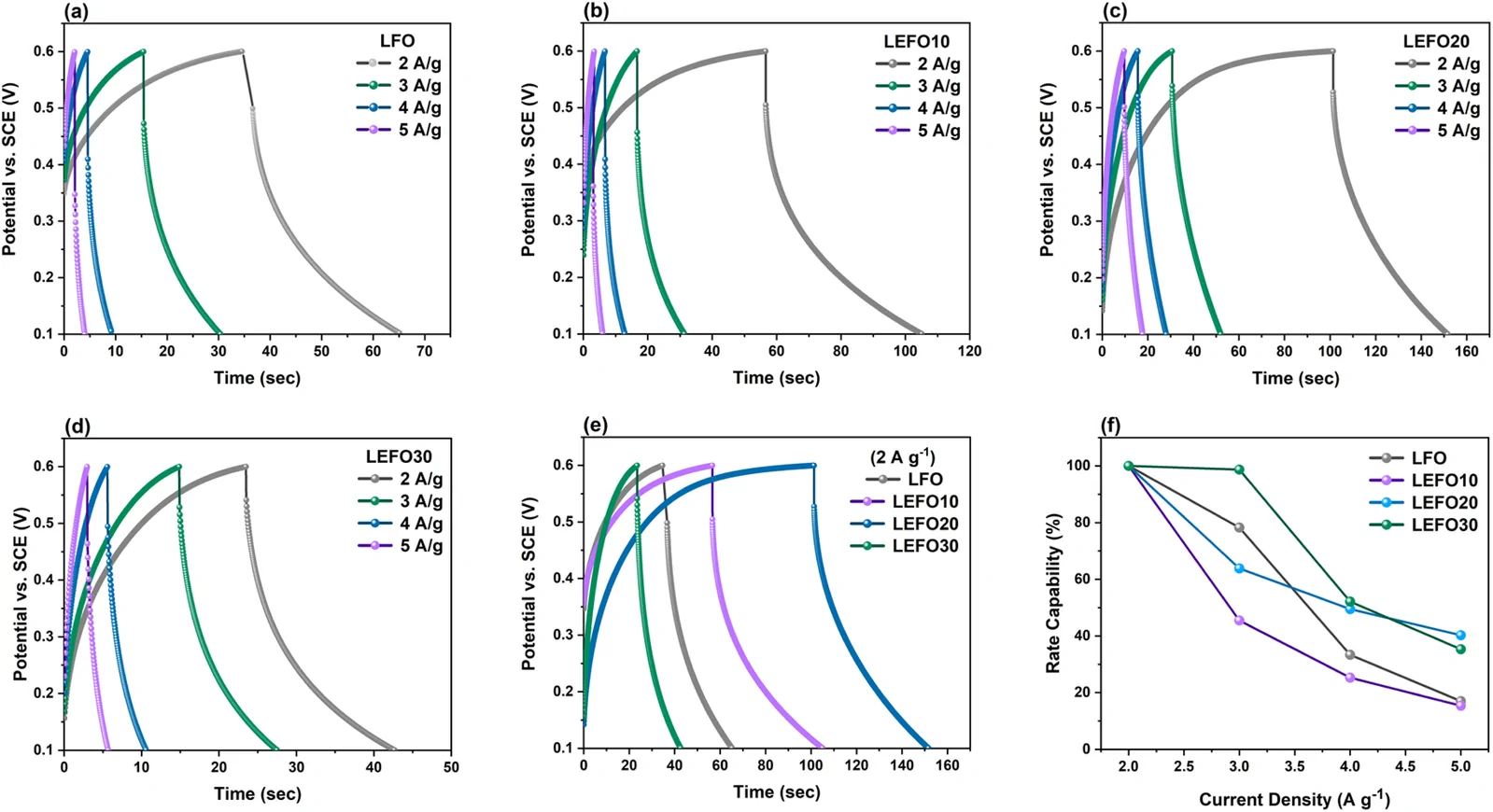
Galvanostatic charge–discharge (GCD) curves of (a) LFO, (b) LEFO10, (c) LEFO20, (d) LEFO30 at different current densities, (e) all working electrodes at 2 A g^−1^, and (f) rate capability *vs.* current density.

The GCD tests were performed in the same potential window (0.1 to 0.6 V) as the cyclic voltammetry and at different current densities (2, 3, 4, and 5 A g^−1^) to evaluate the capacitive behavior, rate capability, and cyclic performance of the fabricated working electrodes. The GCD curves for LFO, LEFO10, LEFO20, and LEFO30 are illustrated in Fig. 11(a–d). In general, linear and symmetrical triangular curves during the galvanostatic charge–discharge cycles are indicative of EDLC-type supercapacitors, whereas the presence of plateaus in the GCD charge–discharge curves indicates the faradaic charge storage mechanism. The non-linear and asymmetrical shape of the curves, as shown in Fig. 11(a–d), suggested the significant contribution of the pseudocapacitive behavior (ion intercalation, surface redox reaction, diffusion-controlled processes) of the working electrodes, which was in line with the observations from the CV curves as well. From the GCD curves, the specific capacitance (*C*_sp_) values were calculated using eqn (6), and they are tabulated in Table 7.6
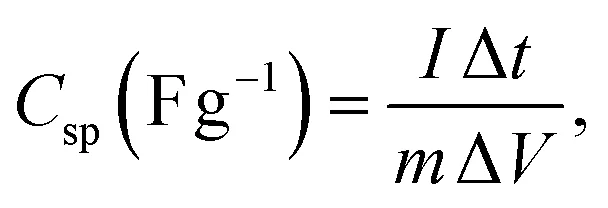
where *C*_sp_ is the specific capacitance, ‘*I’* is the constant current flowing through the electrodes, ‘*m*’ is the mass of the active electrode material, Δ*V* is the potential window, and Δ*t* is the discharge time. For a current density of 2 A g^−1^, the *C*_sp_ values increased from 114.5 F g^−1^ to 194 F g^−1^, and finally to 201.4 F g^−1^ for the LFO, LEFO10, and LEFO20 electrodes. But for the LEFO30 electrode, the *C*_sp_ value decreased to 76.7 F g^−1^, which followed the trend of the *C*_sp_ values obtained from the CV curves using eqn (4). From Fig. 11(a–d), it is observed that with the increase in current density, the non-linear asymmetric GCD curves become nearly linear in shape, suggesting a decrease in pseudocapacitive contribution, *i.e.*, ions start to lose their ability to intercalate into the bulk coating material and are limited to adsorption at the surface of the electrodes.^58^ As such, the discharge times decreased significantly, resulting in a decrease in *C*_sp_ values at high current densities. So, rate capability is an important parameter that describes the capacitance retention of an electrode with the increase in current density. The rate capability of the working electrodes was calculated using eqn (7). LEFO20 indicated the highest rate capability of 40% among all the fabricated electrodes. From the GCD curves shown in Fig. 11(e), it is observed that the *iR*-drop at the start of the discharge cycle is the lowest for LEFO20, which suggests lower equivalent resistance in the LEFO20 electrode. This facilitated the ion diffusion into the electrode material and resulted in a higher charge storage capacity with good rate capability (illustrated in Fig. 11(f)).7
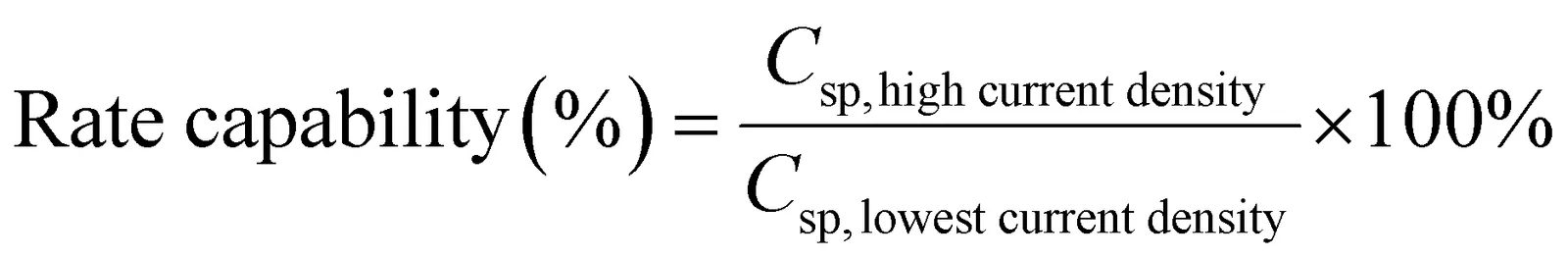


**Table 7:** Specific capacitances (*C*_sp_) of the working electrodes at different current densities from galvanostatic charge–discharge

Current density (A g^−1^)	Specific capacitance, *C*_sp_ (F g^−1^)			
	LFO	LEFO10	LEFO20	LEFO30
2	114.5	194	201.4	76.7
3	89.59	88.2	128.6	75.7
4	38.2	49.1	99.7	40
5	19.5	29.9	81.1	27.1

**Fig. 12:**
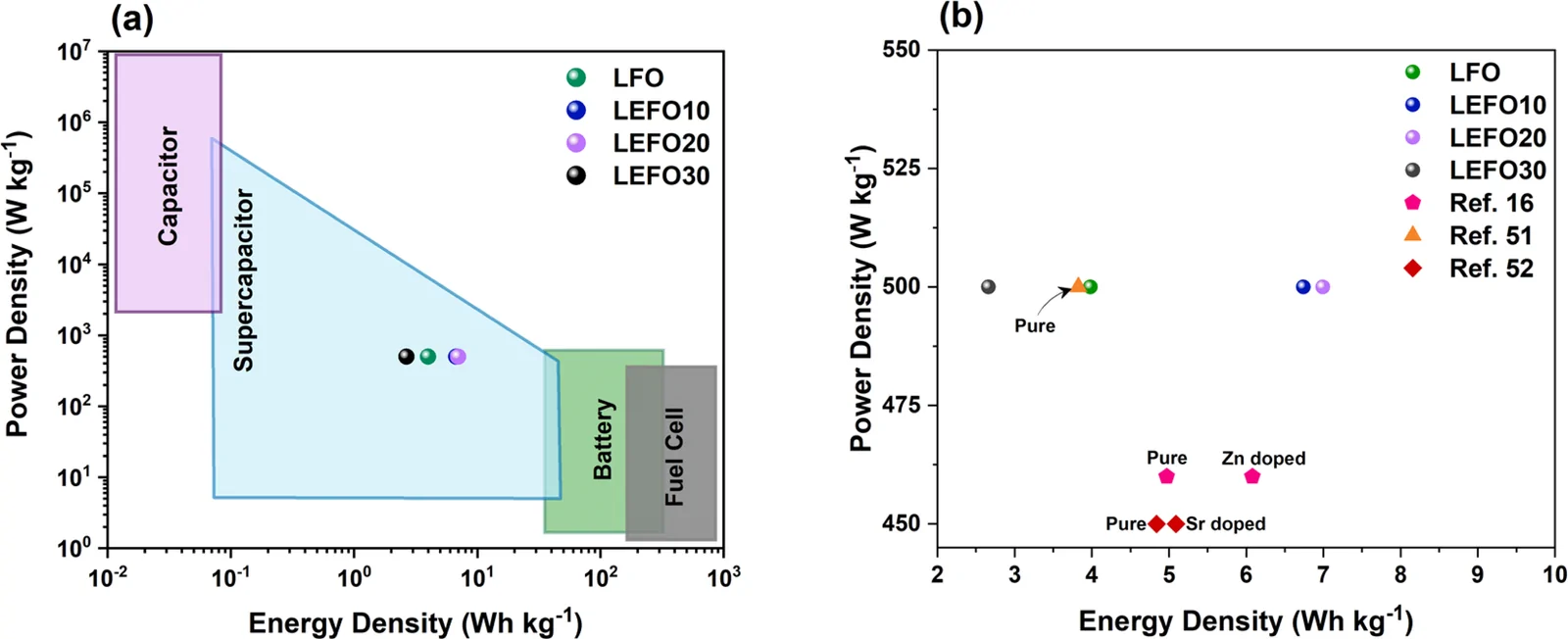
(a) Energy and power densities of the working electrodes in a Ragone plot. (b) Comparison of the current work with reported pristine and doped LaFeO_3_ in a Ragone plot.

Energy and power densities are two other important parameters for analyzing the electrochemical performance of supercapacitors. These are calculated using eqn (8) and (9), and the obtained values are plotted in the Ragone plot (Fig. 12(a)) to suggest its application as a supercapacitor. From Table 8, it can be observed that LEFO20 can store the maximum amount of charge compared to the other electrodes, maintaining a similar charge transfer rate.8
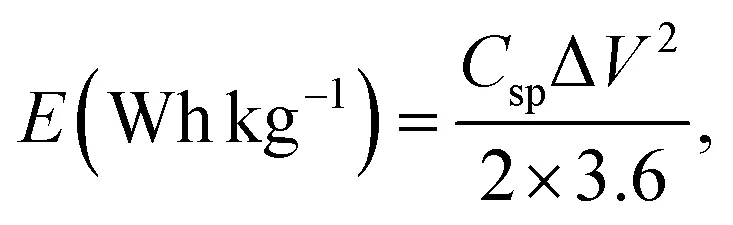
9
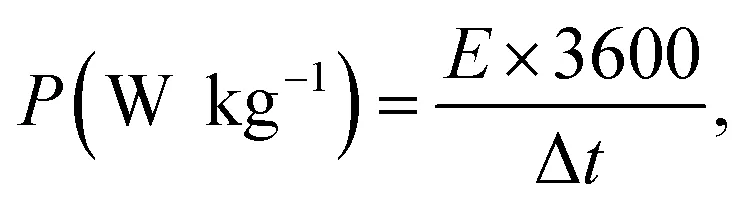
where *C*_sp_ is the specific capacitance, Δ*V* is the potential window, Δ*t* is the discharge time, ‘*E*’ is the energy density, and ‘*P*’ is the power density.

**Table 8:** Energy and power densities of the working electrodes at 2 A g^−1^

	LFO	LEFO10	LEFO20	LEFO30
Energy density (Wh kg^−1^)	3.98	6.74	6.99	2.66
Power density (W kg^−1^)	500	500	500	500

Cyclic stability is another critical electrochemical performance metric for supercapacitors to test for their durability and cycle life. The cyclic performance is analyzed by calculating the capacitance retention and coulombic efficiency of the working electrodes using eqn (10) and (11).10
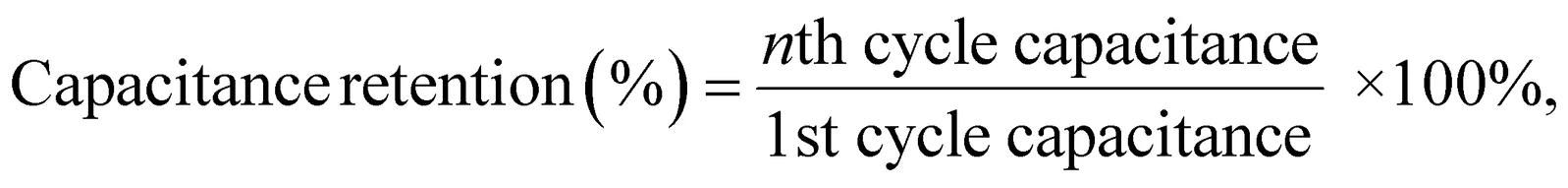
11
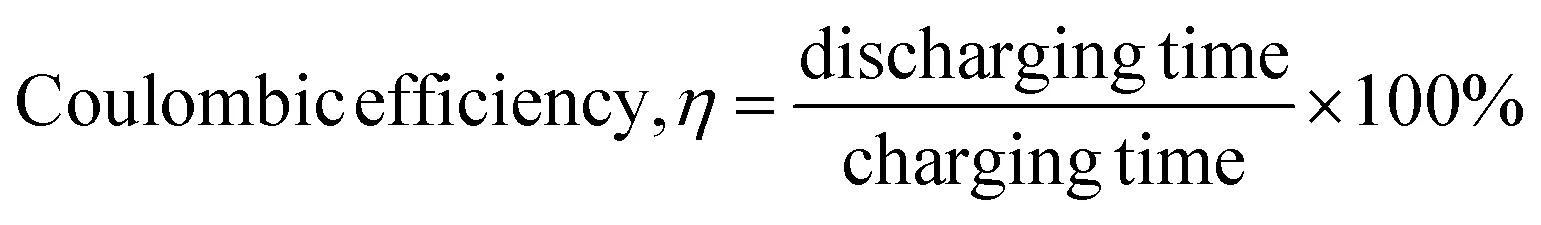


**Fig. 13:**
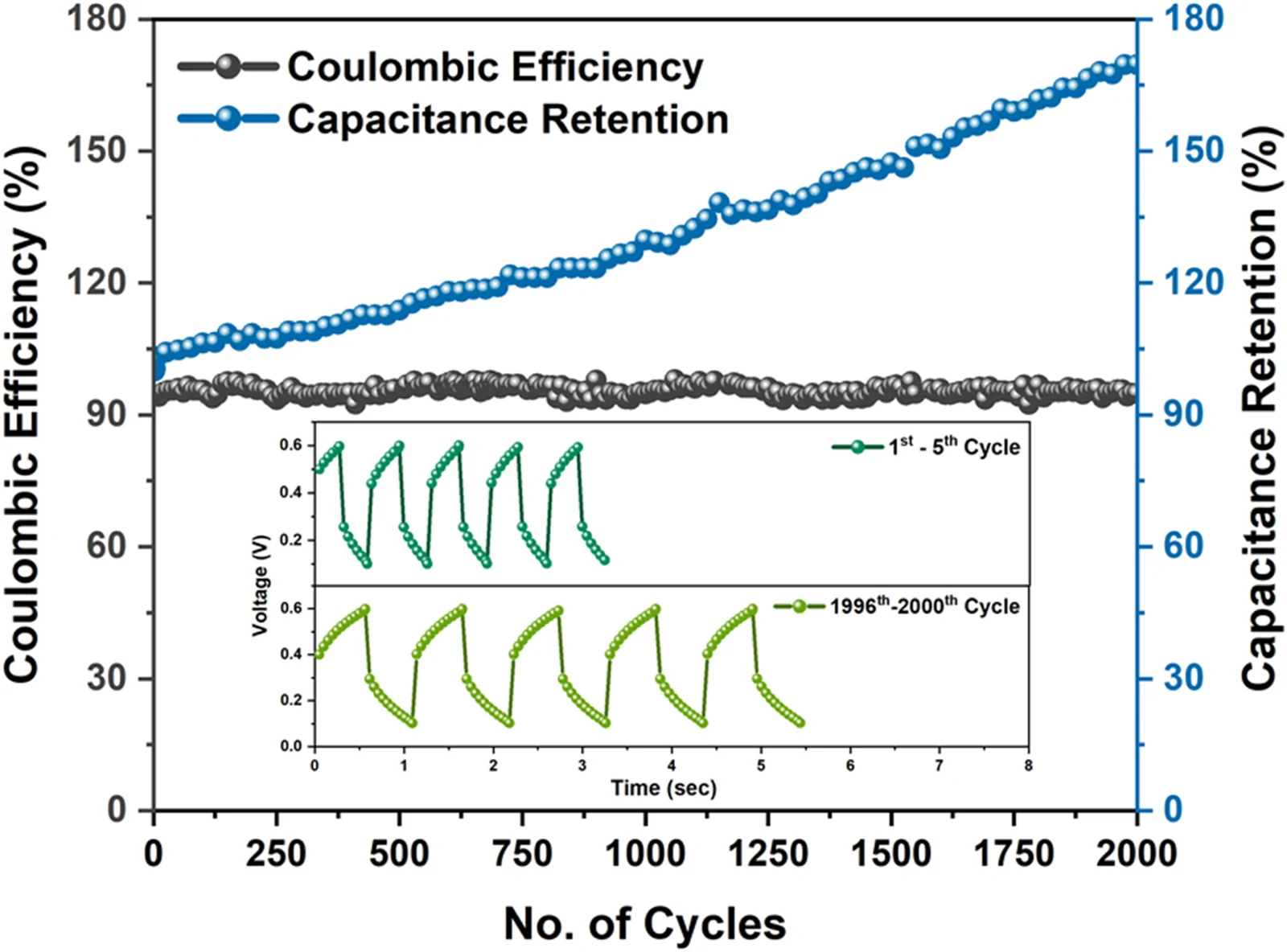
Cyclic stability of the LEFO20 electrode after 2000 cycles of continuous charging and discharging.

The long-term cyclic performance of the LEFO20 working electrode was analyzed by its capacitance retention and coulombic efficiency after 2000 cycles of continuous charging and discharging (GCD) tests at a current density of 20 A g^−1^, and it is illustrated in Fig. 13. The capacitance retention was 171% of the initial capacitance after 2000 cycles of charging and discharging. The increase in specific capacitance (*C*_sp_) values could be attributed to a progressive electrochemical activation process during repeated charge–discharge cycles, *i.e.*, the self-activation of the electrodes in the initial few cycles and the enhancement of the wettability of the electrodes over time.^64^ This behavior was associated with the gradual activation of previously inaccessible electrochemically active sites, particularly those related to defects, as well as improved electrolyte penetration into the electrode structure. The enhanced wettability of the electrode over time facilitated more efficient ion diffusion and increased the utilization of active material, thereby contributing to the observed increase in specific capacitance.^65,66^ However, capacitance values exceeding 100% retention should be interpreted with caution, as they may partly arise from gradual changes at the electrode–electrolyte interface during the initial cycling process.

In conclusion, the electrochemical performance of the Er-substituted system was governed by the combined effects of crystallite size refinement, lattice strain evolution, and defect chemistry. At moderate Er concentrations (20 mol%), controlled lattice distortion facilitated the formation of defects, which enhanced defect-mediated ion transport and improved the availability of electrochemically active sites. Simultaneously, the reduction in crystallite size shortened ion diffusion pathways and promoted more efficient charge transfer kinetics, leading to lower diffusion resistance and enhanced electrochemical activity. However, at high Er substitution levels, the substantial increase in lattice strain introduced structural disorder that could disrupt transport pathways and limit further improvement in the electrochemical performance. The present study focuses on elucidating the intrinsic electrochemical behavior of Er-substituted LaFeO_3_ using a three-electrode configuration, which is widely employed for the fundamental evaluation of electrode materials. Accordingly, the reported specific capacitance, energy density, and power density reflected the intrinsic electrochemical characteristics of the electrode rather than the performance of a practical supercapacitor device. Device-level validation using a two-electrode symmetric or asymmetric configuration could not be performed due to the lack of appropriate fabrication and testing facilities. Such investigations will be pursued in future work to evaluate the practical applicability of the optimized electrode material.

To place the present work in the context of previously reported LaFeO_3_-based supercapacitor electrodes, a comparison of representative literature is summarized in Table 9. The table includes pristine and doped LaFeO_3_ systems, together with their electrochemical performances. This comparison indicated that the Er-substituted LaFeO_3_ developed in the present study exhibited competitive electrochemical performance while providing a clear structure–defect–property correlation through rare-earth-induced defect engineering.

**Table 9:** Comparative study of LaFeO_3_ perovskites and their modifications for enhanced electrochemical performance

Perovskite oxide	Synthesis route	Morphology	Electrolyte	Electrochemical property	Ref.
LaFeO_3_	Room-temp. Electrospinning	Nanofibers	6 M KOH	125.3 F g^−1^ at 5 mV s^−1^, 110.16 F g^−1^ at 2 A g^−1^	67
Sr-doped LaFeO_3_	Solid-state synthesis	Spherical nanoparticles	—	Pure	68
				106 F g^−1^ at 5 mV s^−1^	
				172 F g^−1^ at 2 A g^−1^	
				Sr-doped	
				228 F g^−1^ at 5 mV s^−1^	
				181 F g^−1^ at 2 A g^−1^	
La_0.8_Na_0.2_Fe_0.8_Mn_0.2_O_3_	Modified Pechini	Nanoparticles (NPs)	1 M H_2_SO_4_	56.4 F g^−1^ at 3 mV s^−1^	69
Al-doped LaFeO_3_	Citrate-nitrate auto ignition	Nanoparticles (NPs)	1 M H_2_SO_4_	Pure	20
				200 F g^−1^ at 0.5 mV s^−1^	
				Al-doped	
				260 F g^−1^ at 0.5 mV s^−1^	
Zn-doped LaFeO_3_	Solid-state synthesis	—	—	Pure	19
				235 F g^−1^ at 5 mV s^−1^	
				169 F g^−1^ at 2 A g^−1^	
				Zn-doped	
				141 F g^−1^ at 5 mV s^−1^	
				207 F g^−1^ at 2 A g^−1^	
La_0.8_Nd_0.2_Fe_0.8_Mn_0.2_O_3_	Hydrothermal synthesis	Spherical nanoparticles	3 M KOH	158 F g^−1^ at 50 mV s^−1^	22
La_0.8_Er_0.2_FeO_3_	Sol–gel synthesis	Nanoparticles	1 M KOH	Pure	This work
				219.07 F g^−1^ at 5 mV s^−1^	
				114.5 F g^−1^ at 2 A g^−1^	
				Er-doped	
				288.2 F g^−1^ at 5 mV s^−1^	
				201.4 F g^−1^ at 2 A g^−1^	

## 4. Conclusion

Er^3+^ A-site substitution effectively modulated the defect structure and electrochemical behavior of LaFeO_3_, highlighting the role of rare-earth doping in tuning charge-storage mechanisms. Electrochemical analysis revealed that charge storage was governed by a mixed mechanism dominated by diffusion-controlled intercalation, with a secondary contribution from surface-controlled capacitive processes. Among all compositions, the La_0.8_Er_0.2_FeO_3_ electrode exhibited the best performance due to its improved defect concentration, ion diffusion pathways, reduced particle size, and enhanced electrochemically active surface area. The improved cycling behavior further suggested the structural stability and electrochemical reversibility of the optimized composition. However, the dominance of diffusion-controlled kinetics indicated that the rate performance remained partially limited. Overall, this study highlighted A-site rare-earth substitution as an effective strategy for tuning defect chemistry and improving charge-storage behavior in perovskite oxides. While the present study establishes a clear structure-defect-property relationship, future work involving device-level validation and direct transport measurements will be important for translating these findings into practical applications.

## Author contributions

Tomal Saha: writing – original draft, visualization, methodology, investigation, formal analysis, data curation, conceptualization. Ananya Roy: writing – original draft, visualization, methodology, investigation, formal analysis. Sadman Yasir: writing – original draft, investigation, data curation. Shahid Bin Anik: writing – original draft, investigation, data curation. Md. Fakhrul Islam: writing – review & editing, resources, investigation, funding acquisition. Aninda Nafis Ahmed: writing – review & editing, resources, investigation, formal analysis. Takian Fakhrul: writing – review & editing, supervision, project administration, resources, funding acquisition, methodology, formal analysis, conceptualization.

## Conflicts of interest

The authors declare that they have no known competing financial interests or personal relationships that could have appeared to influence the work reported in this paper.

## Supplementary Material

RA-016-D6RA04225B-s001

## Data Availability

The data that support the findings of this study are available from the corresponding author upon reasonable request. Supplementary information (SI): multi-point EDS spectra of the LFO, LEFO10, LEFO20, and LEFO30 samples; XPS survey spectra; Kramers–Kronig validation of the electrochemical impedance data; XRD peak-shift analysis; and the capacitive and diffusion-controlled charge-storage contributions obtained from Dunn's analysis. See DOI: https://doi.org/10.1039/d6ra04225b.
