# Supplementary material for: Erbium-substituted LaFeO3 perovskite nanoparticles for pseudocapacitive energy storage applications
Source: RSC Adv. 2026 Aug 5;16(39):44939–56. doi: 10.1039/d6ra04225b (PMC13440470; doi:10.1039/d6ra04225b)
Supplement: Supplementary file 1 [file RA-016-D6RA04225B-s001.pdf]

Supplementary Information (SI) for RSC Advances.
This journal is © The Royal Society of Chemistry 2026

Supplementary Information

Erbium-Substituted $LaFeO_3$ Perovskite Nanoparticles for Pseudocapacitive Energy Storage Applications

Authors: Tomal Saha [a]†, Ananya Roy [a], Sadman Yasir [a], Shahid Bin Anik [a], Md. Fakhrul Islam [b], Aninda Nafis Ahmed [c]*, Takian Fakhrul [a]*

Affiliations:

[a] Department of Materials and Metallurgical Engineering, Bangladesh University of Engineering and Technology (BUET), Dhaka, 1000, Bangladesh.

[b] Department of Nanomaterials and Ceramic Engineering, Bangladesh University of Engineering and Technology (BUET), Dhaka, 1000, Bangladesh.

[c] Pilot Plant and Process Development Centre, Bangladesh Council of Scientific and Industrial Research (BCSIR), Dhaka 1205, Bangladesh.

*** Corresponding authors:** Takian Fakhrul (takianf@mme.buet.ac.bd) and Aninda Nafis Ahmed (a_nafis_ahmed@bcsir.gov.bd)

Supplementary information captions:

Figure S1: EDS spectra from multiple points of the LFO sample
Figure S2: EDS spectra from multiple points of the LEFO10 sample
Figure S3: EDS spectra from multiple points of the LEFO20 sample
Figure S4: EDS spectra from multiple points of the LEFO30 sample
Figure S5: XPS survey spectra of LFO and LEFO20 samples
Figure S6: % Residual errors of the obtained impedimetric data after validation by the Kramer-Kronig Test

Table S1: Peak shifts of the major peaks in the XRD pattern of the synthesized nanoparticles
Table S2: Capacitive and Diffusion-controlled contributions to charging storing at the electrodes

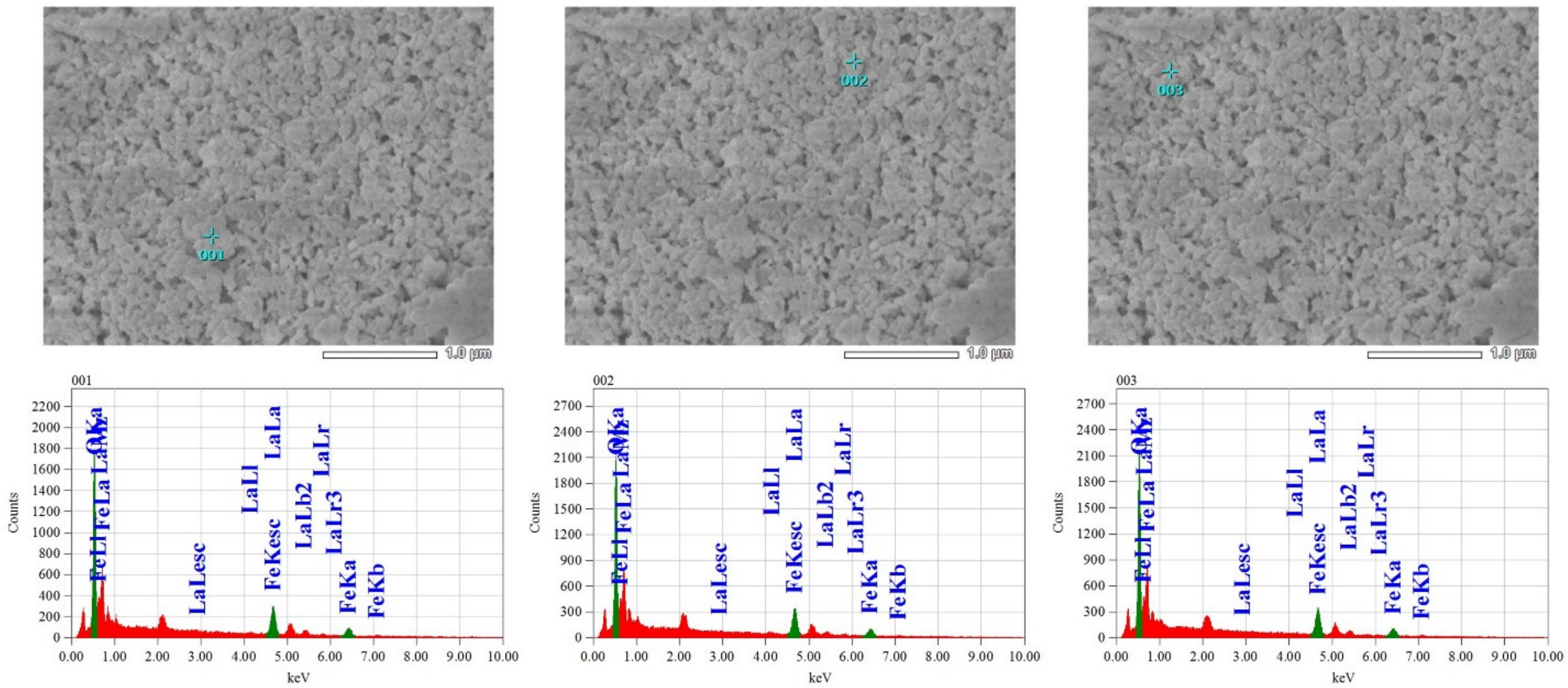

***Figure S1**: EDS spectra from multiple points of the LFO sample*

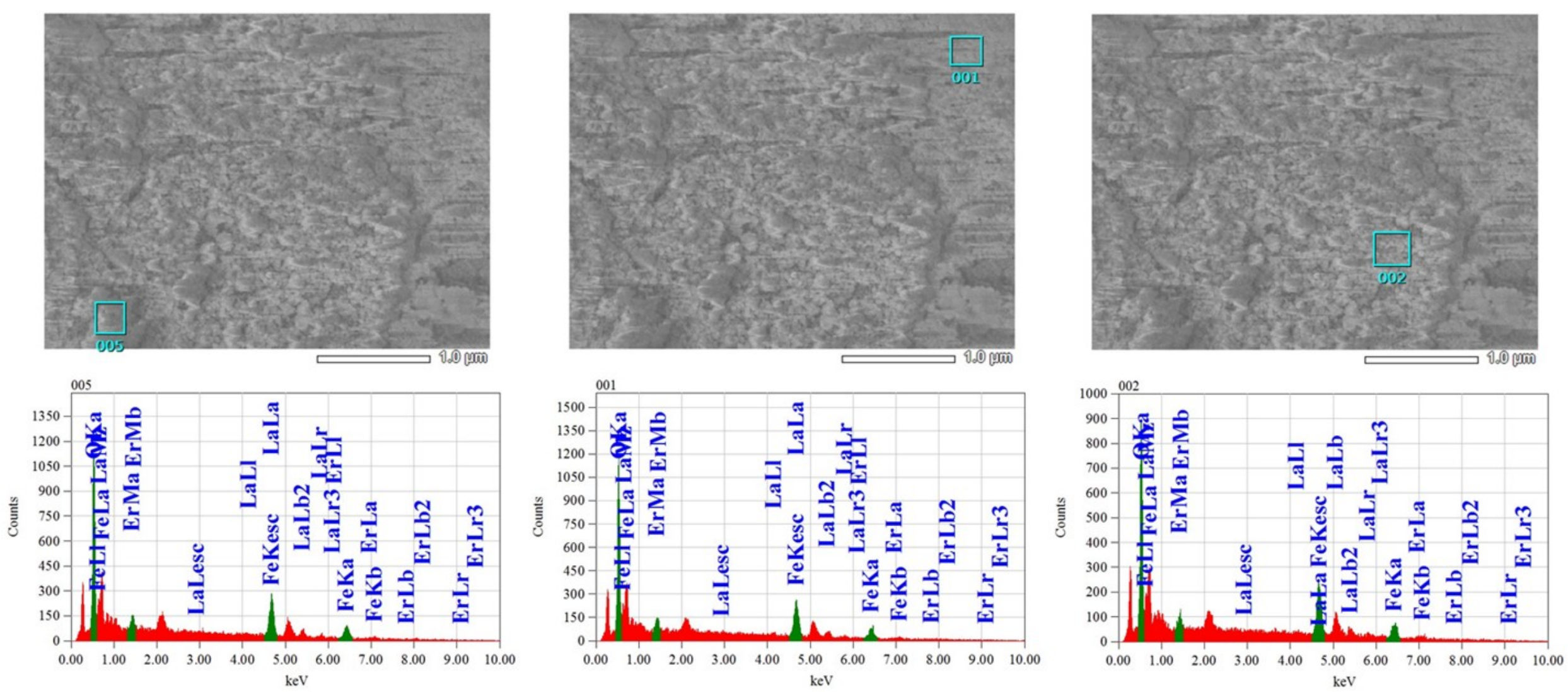

***Figure S2**: EDS spectra from multiple points of the LEFO10 sample*

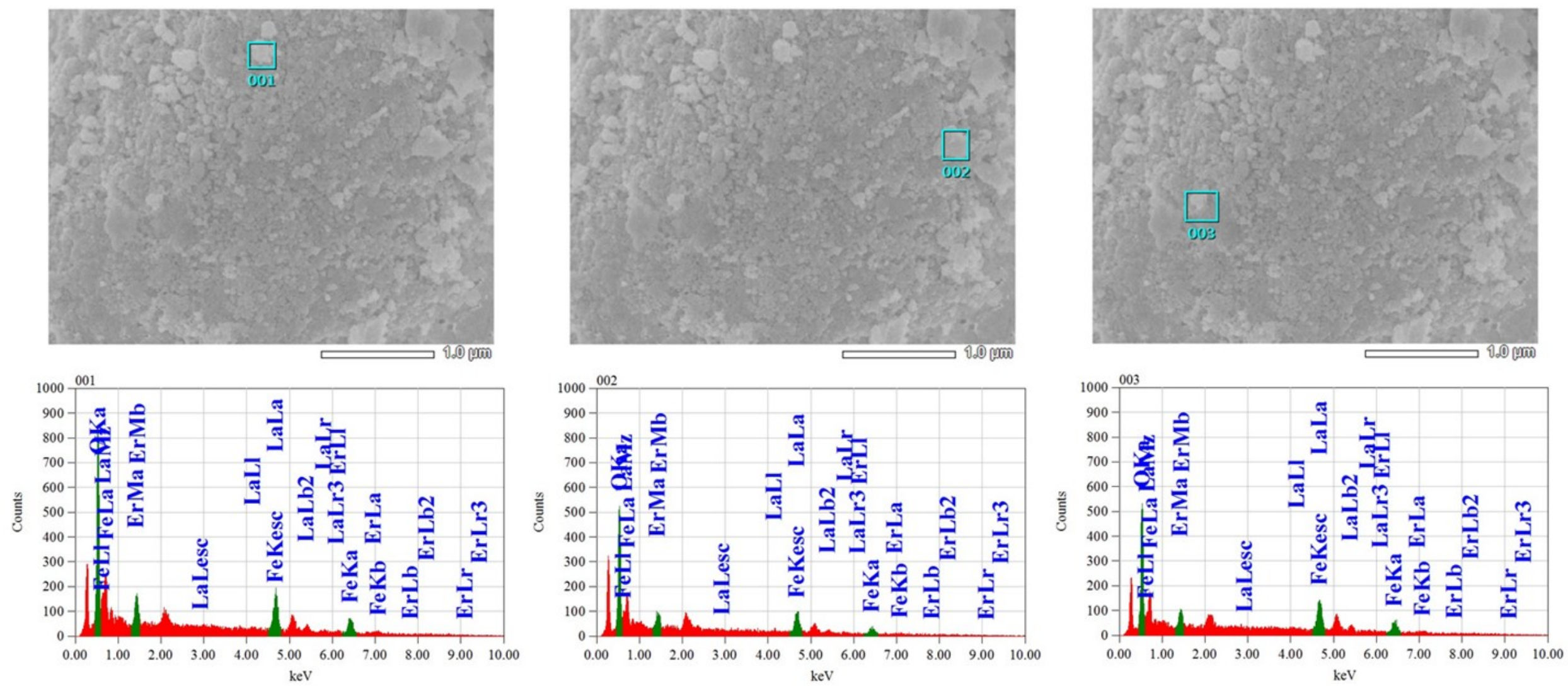

Figure S3: *EDS spectra from multiple points of the LEFO20 sample*

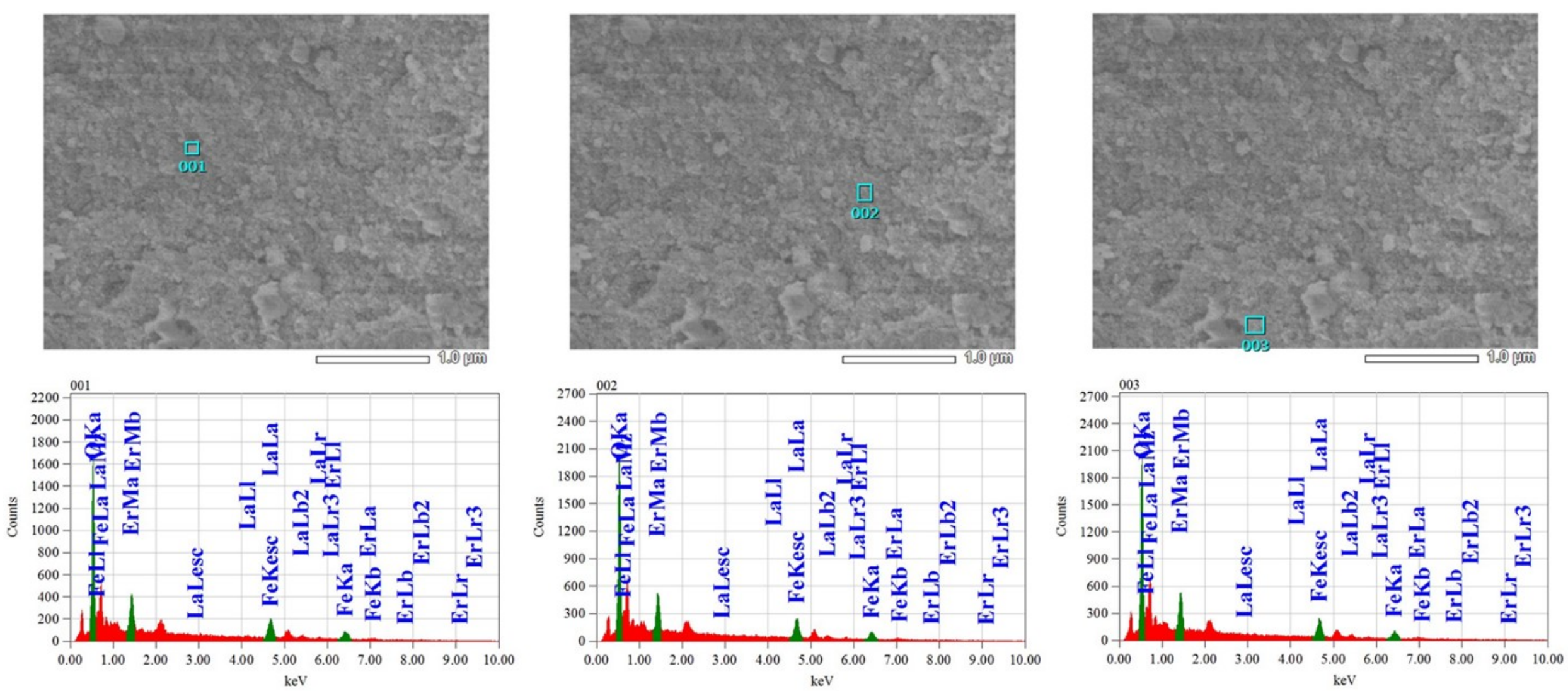

Figure S4: *EDS spectra from multiple points of the LEFO30 sample*

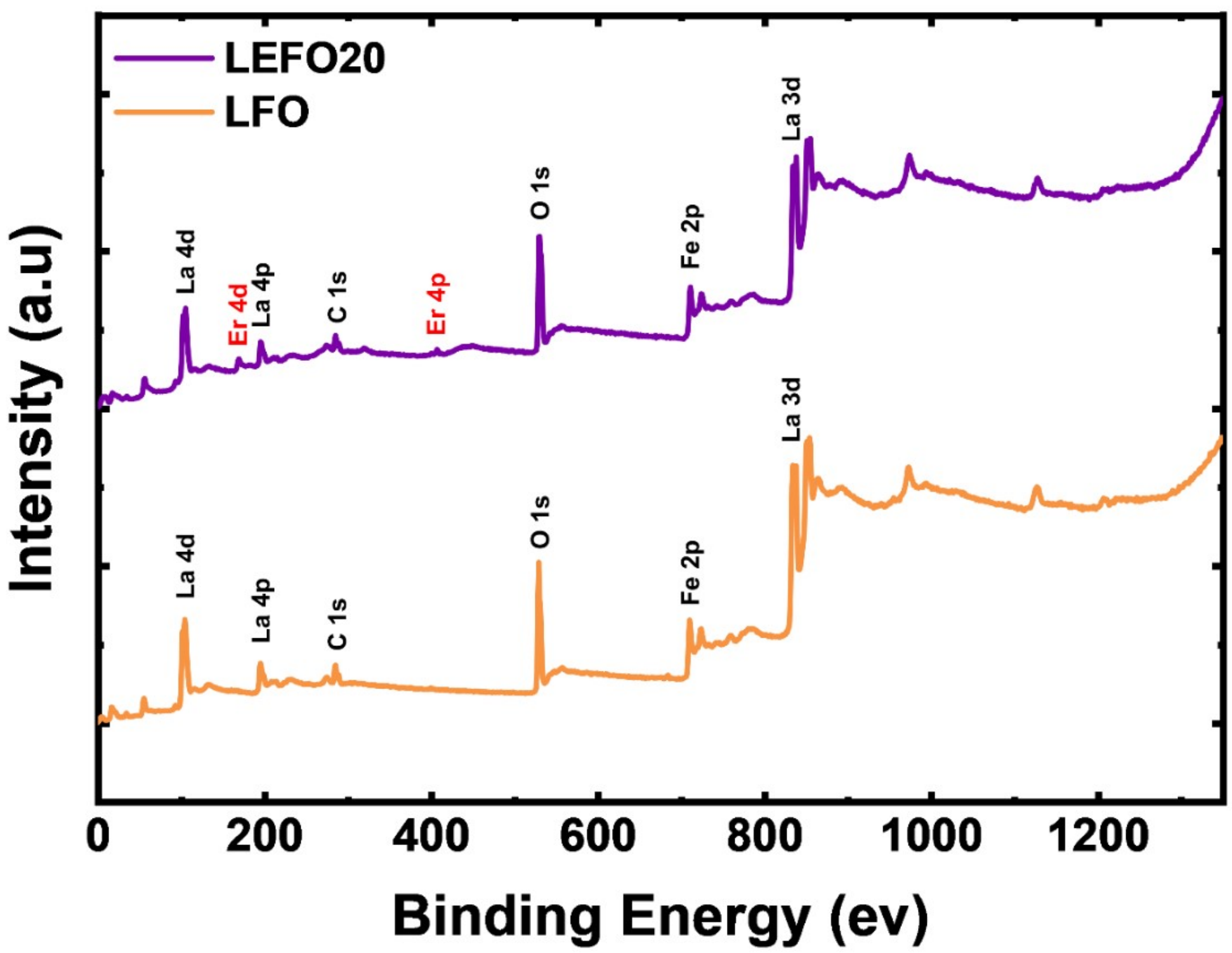

Figure S5: *XPS survey spectra of LFO and LEFO20 samples*

Electrochemical Impedance Spectroscopy:

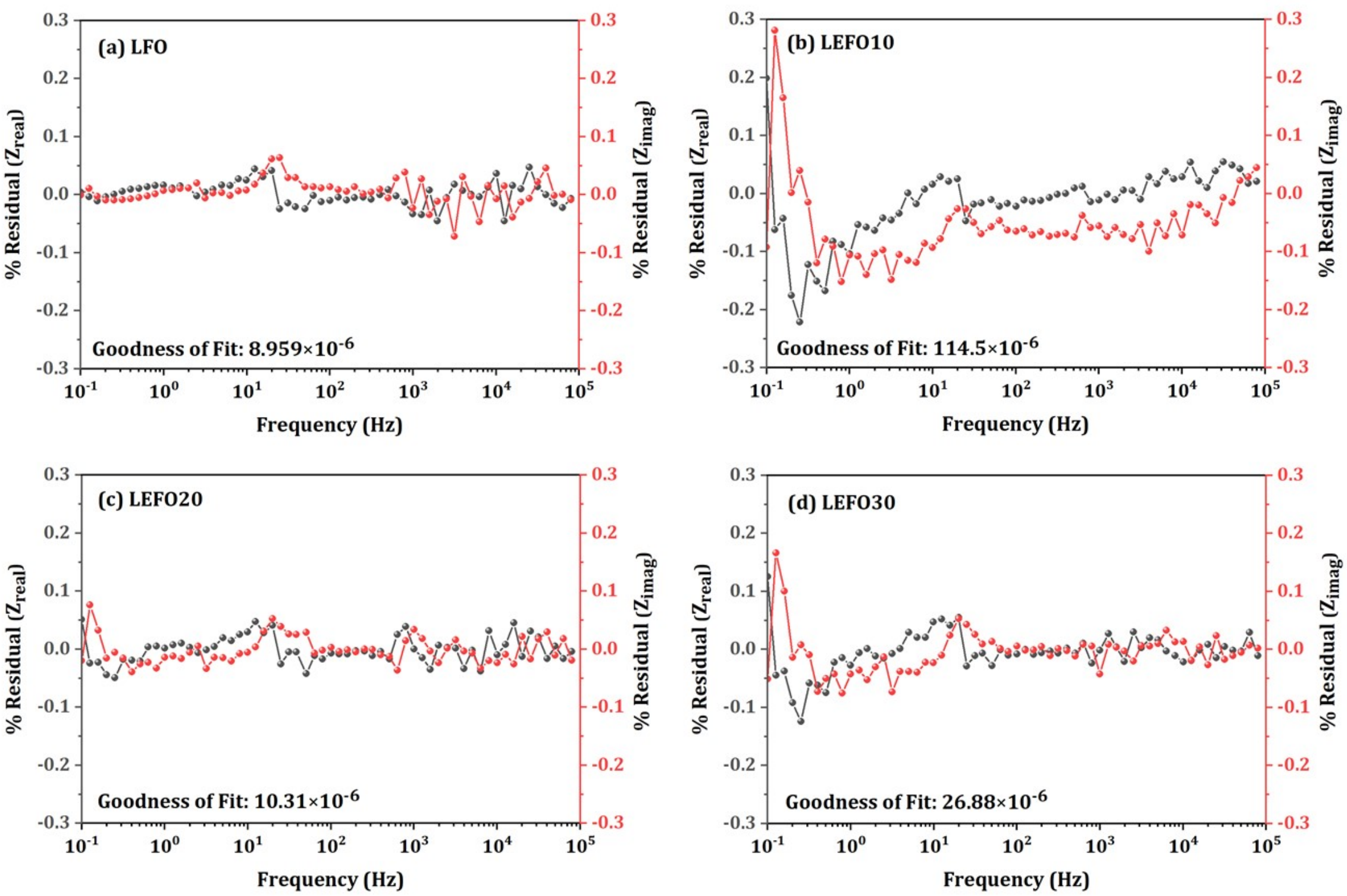

Figure S6: *% Residual errors of the obtained impedimetric data after validation by the Kramer-Kronig Test*

The Warburg impedance (Z_w) is a combination of R_w and C_w in series, where both the parameters are frequency dependent and can be expressed as Equation (S1).

$$Z_w(\omega) = \sigma\frac{1-j}{\sqrt{\omega}} \ldots\ldots\ldots\ldots\ldots\ldots (S1)$$

Where σ is the Warburg coefficient in Ω-$s^{0.5}$, ω is the frequency. The Warburg impedance (Z_w) can be converted to the Warburg admittance (Y_w) and can be written as Equation (2). From the fitted parameters obtained from EChem Analyst and Equation (S2), the 'σ' value is calculated.

$$Y_w(\omega) = \frac{\sqrt{\omega}}{2\sigma}(1+j) \ldots\ldots\ldots\ldots\ldots\ldots (S2)$$

Table S1: *Peak shifts of the major peaks in the XRD pattern of the synthesized nanoparticles*

(hkl)	2θ (degrees)			
	**LFO**	**LEFO10**	**LEFO20**	**LEFO30**
(101)	22.5517	22.5612	22.5733	22.6825
(121)	32.1714	32.2054	32.2595	32.3523
(220)	39.6035	39.6475	39.6875	39.7462
(202)	46.0880	46.1736	46.3016	46.4704
(123)	57.4289	57.5150	57.6736	57.8776
(242)	67.3595	67.5153	67.6173	67.7556

Table S2: *Capacitive and Diffusion-controlled contributions to charge storing at the electrodes*

**Scan Rate (mV/s)**	**LFO**	**LEFO10**	**LEFO20**	**LEFO30**	**LFO**	**LEFO10**	**LEFO20**	**LEFO30**
	**Capacitive Contribution (%)**				**Diffusion-controlled Contribution (%)**			
**5**	13.70	13.97	15.04	10.49	86.30	86.03	84.96	89.51
**10**	18.34	18.67	20.02	14.22	81.66	81.33	79.98	85.78
**20**	24.10	24.51	26.14	18.99	75.90	75.49	73.86	81.01
**50**	33.43	33.92	35.89	27.04	66.57	66.08	64.11	72.96
**100**	41.53	42.06	44.18	34.39	58.47	57.94	55.82	65.61
